# Global, Regional, and National Burden of Myocarditis From 1990 to 2017: A Systematic Analysis Based on the Global Burden of Disease Study 2017

**DOI:** 10.3389/fcvm.2021.692990

**Published:** 2021-07-02

**Authors:** Xiqiang Wang, Xiang Bu, Linyan Wei, Jing Liu, Dandan Yang, Douglas L. Mann, Aiqun Ma, Tomohiro Hayashi

**Affiliations:** ^1^Department of Cardiovascular Medicine, Shaanxi Provincial People's Hospital, Xi'an, China; ^2^Department of Cardiovascular Medicine, The Third Affiliated Hospital of Xi'an Jiaotong University, Xi'an, China; ^3^Department of Respiratory and Critical Care Medicine, The First Affiliated Hospital of Xi'an Jiaotong University, Xi'an, China; ^4^Department of Cardiovascular Medicine, The First Affiliated Hospital of Xi'an Jiaotong University, Xi'an, China; ^5^Department of Cardiovascular Medicine, The Second Affiliated Hospital of Zhejiang University, Hangzhou, China; ^6^Cardiovascular Division, Department of Medicine, Center for Cardiovascular Research, Washington University School of Medicine, St. Louis, MO, United States; ^7^Division of Cardiovascular Medicine, Department of Internal Medicine, Kobe University Graduate School of Medicine, Kobe, Japan

**Keywords:** myocarditis, Global Burden of Disease, incidence, death, disability-adjusted life years

## Abstract

**Objective:** The global trends in myocarditis burden over the past two decades remain poorly understood and might be increasing during the coronavirus disease 2019 (COVID-19) worldwide pandemic. This study aimed to provide comprehensive estimates of the incidence, mortality, and disability-adjusted life years (DALYs) for myocarditis globally from 1990 to 2017.

**Methods:** Data regarding the incidence, mortality, DALY, and estimated annual percentage change (EAPC) between 1990 and 2017 for myocarditis worldwide were collected and calculated from the 2017 Global Burden of Disease study. We additionally calculated the myocarditis burden distribution based on the Socio-Demographic Index (SDI) quintile and Human Development Index (HDI).

**Results:** The incidence cases of myocarditis in 2017 was 3,071,000, with a 59.6% increase from 1990, while the age-standardized incidence rate (ASIR) was slightly decreased. The number of deaths due to myocarditis increased gradually from 27,120 in 1990 to 46,490 in 2017. The middle SDI quintile showed the highest number of myocarditis-related deaths. On the contrary, the global age-standardized death rate (ASDR) decreased with an overall EAPC of −1.4 [95% uncertainty interval (UI) = −1.8 to −1.0]. Similar to ASDR, the global age-standardized DALY rate also declined, with an EAPC of −1.50 (95% UI = −2.30 to −0.8) from 1990 to 2017. However, there was a 12.1% increase in the number of DALYs in the past 28 years; the middle SDI and low-middle SDI quintiles contributed the most to the DALY number in 2017. We also observed significant positive correlations between the EPAC of age-standardized rate and HDI for both death and DALY in 2017.

**Conclusions:** Globally, the ASIR, ASDR, and age-standardized DALY rate of myocarditis decreased slightly from 1990 to 2017. The middle SDI quintile had the highest level of ASIR, ASDR, and age-standardized DALY rate, indicating that targeted control should be developed to reduce the myocarditis burden especially based on the regional socioeconomic status. Our findings also provide a platform for further investigation into the myocarditis burden in the era of COVID-19.

## Introduction

Cardiovascular disease is a major contributor to disease burden and death globally (1). Myocarditis is a cause of cardiovascular disease that primarily manifests as chest pain, sudden death, and heart failure. There were 353,700 [95% uncertainty index (UI): 339,500 to 370,600] deaths globally due to myocarditis and cardiomyopathy in 2015, representing a considerable public health problem (2, 3). It was also ranked as the third leading cause of sudden cardiac death in competitive athletes reported by the American Heart Association and the American College of Cardiology, and it was considered the most common known cause of dilated cardiomyopathy in children <18 years of age (4).

However, the actual burden of myocarditis is challenging to determine and is likely under-reported, and the burden of myocarditis is either limited to the local scope or does not include an analysis of regional and temporal variations (5, 6). The fundamental knowledge of the incidence, mortality, or disability-adjusted life years (DALYs) for myocarditis, which is used to appropriately guide efforts in improving cardiovascular health at national levels, remains inadequate across the world. Severe acute respiratory syndrome coronavirus 2 (SARS-CoV-2) infection can induce myocardial injury and myocarditis (7–9). In the current worldwide pandemic of coronavirus disease 2019 (COVID-19), the number, incidence, and/or mortality of myocarditis might be increasing. Therefore, fundamental data on the burden of myocarditis before the COVID-19 pandemic is valuable to investigate the impact of COVID-19 on the prospective myocarditis burden.

The remaining knowledge gap can be filled by a systematic evaluation of available data from the Global Burden of Disease (GBD) database that can provide an opportunity for timely and transparent estimates of disease incidence, mortality, DALY, and other metrics to describe the disease burden at the geographic scales of global, regional, and national levels. So far, a large number of articles using the GBD database have been published and serve to improve health policies (10–12).

In the present study, we used the GBD 2017 database to describe the incidence, mortality, and DALY burden of myocarditis by region, age, and sex in 2017 and to evaluate their temporal trends from 1990 to 2017 across the world. Furthermore, we calculated the myocarditis burden distribution based on the Socio-Demographic Index (SDI) quintile and Human Development Index (HDI). The results of this study will help to facilitate the development of global responses that can support the health care system in improving cardiovascular health globally.

## Methods

### Study Data

Detailed data on the incidence, mortality, DALY, and the age-standardized rate (ASR) for myocarditis from 1990 to 2017 were extracted from the Global Health Data Exchange (GHDx) query tool (http://ghdx.healthdata.org/gbd-results-tool) (13). The overall burden of disease was assessed using the DALY, a time-based measure that combines years of life lost due to premature mortality and years of life lost due to time lived in a state of less than full health or years of healthy life lost due to disability (14). The general methods for the GBD study and the methods for estimating myocarditis disease burden have been detailed in a previous study (15). Supplementary Table 1 delineates the International Classification of Diseases 10 (ICD-10) codes and subgroups that embody the GBD myocarditis cause code (B.2.6.1).

Information regarding the sex and age was also retrieved to evaluate their influence on the global burden of myocarditis. To further characterize the global burden of myocarditis, these detailed data were reanalyzed on three levels based on the SDI (16) quintiles, 21 GBD regions, and 195 countries and territories. The five SDI quintiles (high, high-middle, middle, low-middle, and low levels) are based on the SDI, a summary measurement of a region's socio-demographic development status based on total fertility rate, per capita income, and educational level (17). A detailed methodology regarding the calculation of SDI cutoff points is reported elsewhere (1, 18). We then characterized the distribution of the global burden of myocarditis according to the 21 GBD regions. Finally, the annual changes in the incidence rates, mortality rates, and DALY rates in 195 countries and territories over the same time period were described using tables and world maps. HDI (19) is a statistic composite index of life expectancy, education, and per capita income indicators, which is used to rank countries into four tiers of human development. The HDI data in 2017 provided by the World Bank (20) were also extracted by our group to evaluate the relationship between myocarditis disease burden and national development status.

### Statistical Analysis

To compare the myocarditis disease burden of the population between the different demographic structures or within same population in which age profiles changed between different time points, the parameters of age-standardized incidence rate (ASIR), age-standardized death rate (ASDR), and age-standardized DALY rate were used in this study. ASR (per 100,000 population) is equal to the sum of the product of the specific age ratio (*a*_*i*_) in age group *i* and the number [or weight (*w*_*i*_)] of the selected reference standard population group *i* divided by the sum of the number (or weight) of the standard population, i.e., $\text{ASR}=\frac{\overset{A}{\underset{i=1}{\sum }}a_{i}\;w_{i}}{\overset{A}{\underset{i=1}{\sum }}w_{i}}\times \;100,000.$

More importantly, the trend of the ASR can be used as a good surrogate for the rate of incidence, deaths, and DALYs with a shifting pattern in a certain population, and the estimated annual percentage change (EAPC) can serve as a measurement change of ASR trend in a time interval (21). Consequently, a regression line was fitted into the natural logarithm of the rates: *y* = α + β*x* + ϵ, where *y* represents ln ASR and *x* refers to the calendar year. EAPC = 100 × (exp(β) − 1) and its 95% uncertainty interval (UI) can also be obtained from the regression model (21). If the EAPC estimation and its lower limit of 95% UI are both positive, the ASR is considered to be in an increasing trend. Conversely, if the EAPC estimation and its upper limit of 95% UI are both negative, the ASR is in a downward trend. If the above conditions are not met or the estimated UI overlap, the ASR is deemed to be stable. The R program (version 3.6.3, R Core Team) was used to perform the statistical analysis and draft the graphs. Differences were considered significant at a *p*-value of < 0.05.

## Results

### The Incidence Burden of Myocarditis

#### Trends of Myocarditis Incidence From 1990 to 2017

**Figure 1:**
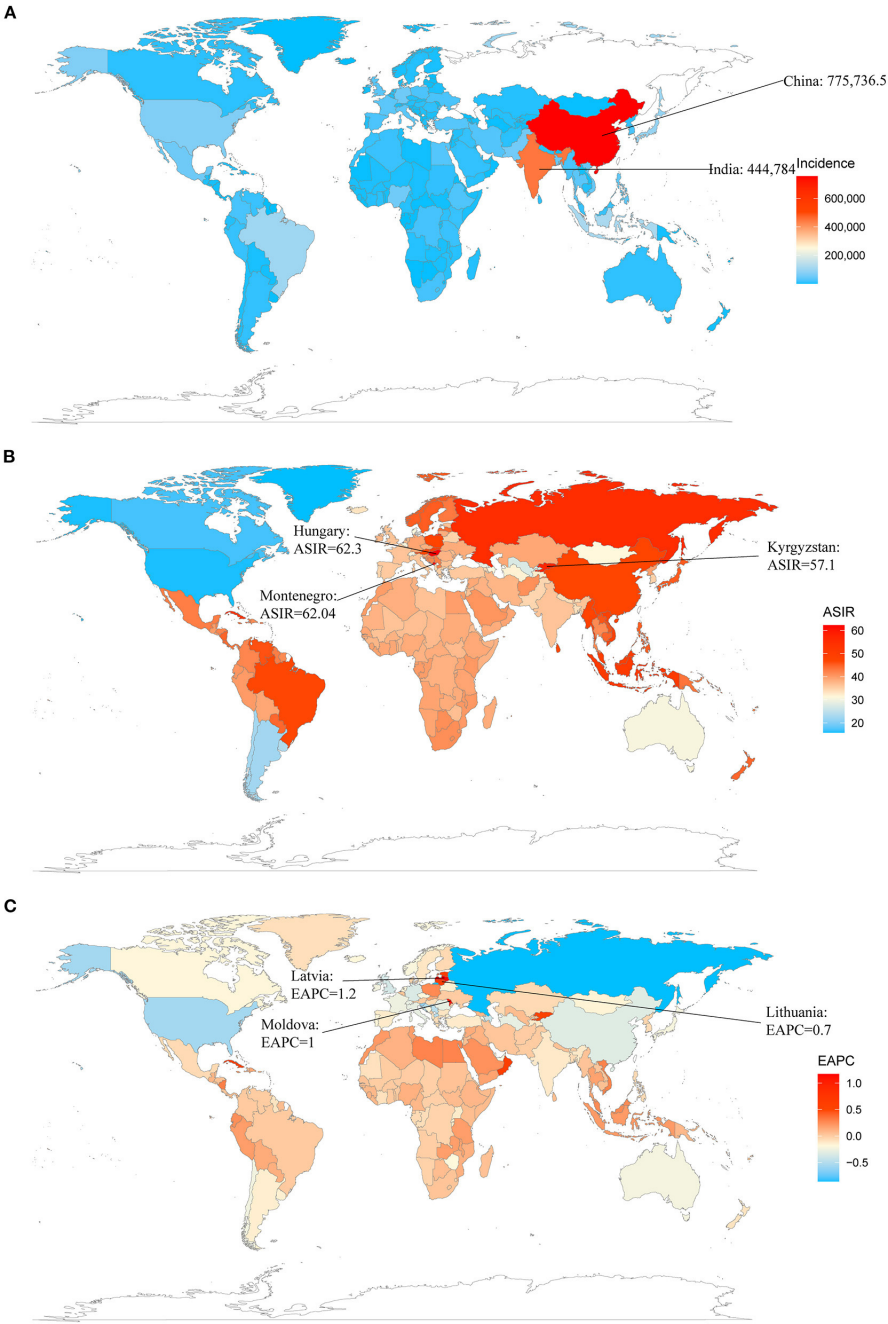
The global incidence burden of myocarditis in 195 countries and territories. **(A)** The absolute number of myocarditis incidence cases in 2017. **(B)** The ASIR of myocarditis in 2017. **(C)** The EAPC of myocarditis ASIRs between 1990 and 2017. ASIR, age-standardized incidence rate; EAPC, estimated annual percentage change.

Globally, the incidence number of myocarditis was 3,071,000 (95% UI = 2,745,000–3,071,000) in 2017, with a 59.6% increase from 1,925,000 (95% UI = 1,741,000–2,164,000) in 1990 (Table 1, Supplementary Table 2, and Figure 1A). In addition, the ASIR decreased globally from 1990 to 2017 globally (41.0 per 100,000 persons in 1990 vs. 39.2 per 100,000 persons in 2017) (Table 1 and Figures 1B,C).

**Table 1:** The incidence cases and age-standardized incidence rate of myocarditis between 1990 and 2017 and its temporal trends from 1990 to 2017.

	**1990**		**2017**		**1990–2017**
**Characteristics**	**Incidence cases No. × 10^**4**^ (95% UI)**	**ASIR per 100,000 No. (95% UI)**	**Incident cases No. × 10^**4**^ (95% UI)**	**ASIR per 100,000 No. (95% UI)**	**EAPC No. (95% CI)**
Global	192.5 (171.4–216.4)	41.0 (36.1–46.1)	307.1 (274.5–307.1)	39.21 (35.1–39.2)	0.0 (−0.6–0.5)
Sex					
Female	94.0 (83.7–105.6)	39.0 (34.8–43.9)	151.8 (135.7–171.3)	37.5 (33.5–42.1)	−0.2 (−0.7–0.2)
Male	98.5 (87.6–110.5)	43.0 (38.5–48.4)	155.3 (138.7–175.0)	41.0 (36.8–46.1)	−0.1 (−0.6–0.4)
Andean Latin America	1.1 (1.0–1.2)	38.00 (33.98–42.99)	2.3 (2.1–2.6)	40.01 (35.72–45.12)	0.2 (−0.3–0.8)
Australasia	0.8 (0.7–0.9)	33.78 (30.34–38.09)	1.3 (1.1–1.4)	32.76 (29.38–36.72)	0.1 (−0.4–0.7)
Caribbean	1.3 (1.2–1.5)	43.58 (38.92–49.05)	2.3 (2.0–2.5)	45.87 (41.01–51.64)	0.1 (0.1–0.2)
Central Asia	2.0 (1.8–2.2)	34.28 (30.74–38.72)	2.7 (2.4–3.1)	33.49 (30.02–37.66)	0.0 (−0.8–0.8)
Central Europe	6.1 (5.4–6.8)	44.87 (40.2–50.45)	7.1 (6.3–8.0)	44.22 (39.71–49.48)	−0.1 (−1.0–0.8)
Central Latin America	5.2 (4.6–5.8)	42.65 (38.18–48.04)	10.5 (9.3–11.8)	42.73 (38.3–48.1)	0.0 (−0.2–0.3)
Central Sub-Saharan Africa	1.4 (1.2–1.6)	38.46 (34.4–43.37)	3.1 (2.7–3.5)	40.4 (36.0–45.59)	0.1 (−0.6–0.8)
East Asia	53.9 (47.9–60.3)	49.8 (44.63–55.85)	79.2 (70.6–89.6)	46.72 (41.81–52.39)	−0.1 (−1.2–1.0)
Eastern Europe	14.4 (12.9–16.2)	56.9 (51.12–63.76)	13.7 (12.2–15.4)	49.8 (44.51–55.82)	−1.0 (−2.2–0.1)
Eastern Sub-Saharan Africa	4.7 (4.1–5.3)	38.11 (33.94–43.02)	10.1 (8.9–11.5)	39.1 (34.9–44.04)	0.1 (−0.4–0.7)
High-income Asia Pacific	8.4 (7.5–9.5)	44.35 (39.69–49.69)	12.0 (10.7–13.6)	42.18 (37.81–47.26)	−0.1 (−1.6–1.4)
High-income North America	5.8 (5.1–6.6)	17.82 (15.76–20.25)	7.8 (7.1–8.6)	16.18 (14.86–17.68)	−0.1 (−2.8–2.6)
North Africa and Middle East	9.1 (8.1–10.2)	37.2 (33.19–41.79)	19.5 (17.3–22.0)	38.29 (34.22–43.13)	0.1 (−0.6–0.8)
Oceania	0.2 (0.2–0.2)	42.58 (38.14–48.22)	0.4 (0.4–0.5)	43.45 (38.83–49.05)	0.0 (−0.7–0.8)
South Asia	29.8 (26.4–33.6)	36.69 (32.76–41.24)	55.5 (49.4–62.8)	35.82 (32.04–40.48)	0.1 (0.0–0.1)
Southeast Asia	16.5 (14.6–18.6)	45.8 (41.07–51.62)	29.5 (26.3–33.4)	47.57 (42.58–53.69)	0.1 (−0.8–1.0)
Southern Latin America	1.1 (1.0–1.2)	23.01 (20.48–25.95)	1.7 (1.5–1.9)	22.65 (20.13–25.57)	0.0 (−1.9–1.9)
Southern Sub-Saharan Africa	1.6 (1.4–1.7)	39.88 (35.66–44.85)	2.7 (2.4–3.0)	40.4 (36.00–45.59)	0.0 (−0.5–0.5)
Tropical Latin America	5.6 (5.0–6.3)	46.25 (41.40–52.00)	10.7 (9.5–12.0)	46.64 (41.75–52.55)	0.0 (−0.2–0.2)
Western Europe	19.0 (17.0–21.4)	56.9 (51.12–63.76)	24.1 (21.5–27.2)	36.99 (33.2–41.48)	0.0 (−0.5–0.4)
Western Sub-Saharan Africa	4.9 (4.4–5.5)	37.51 (33.49–42.45)	11.1 (9.8–12.5)	38.31 (34.22–43.26)	0.1 (−0.4–0.6)

**Figure 2:**
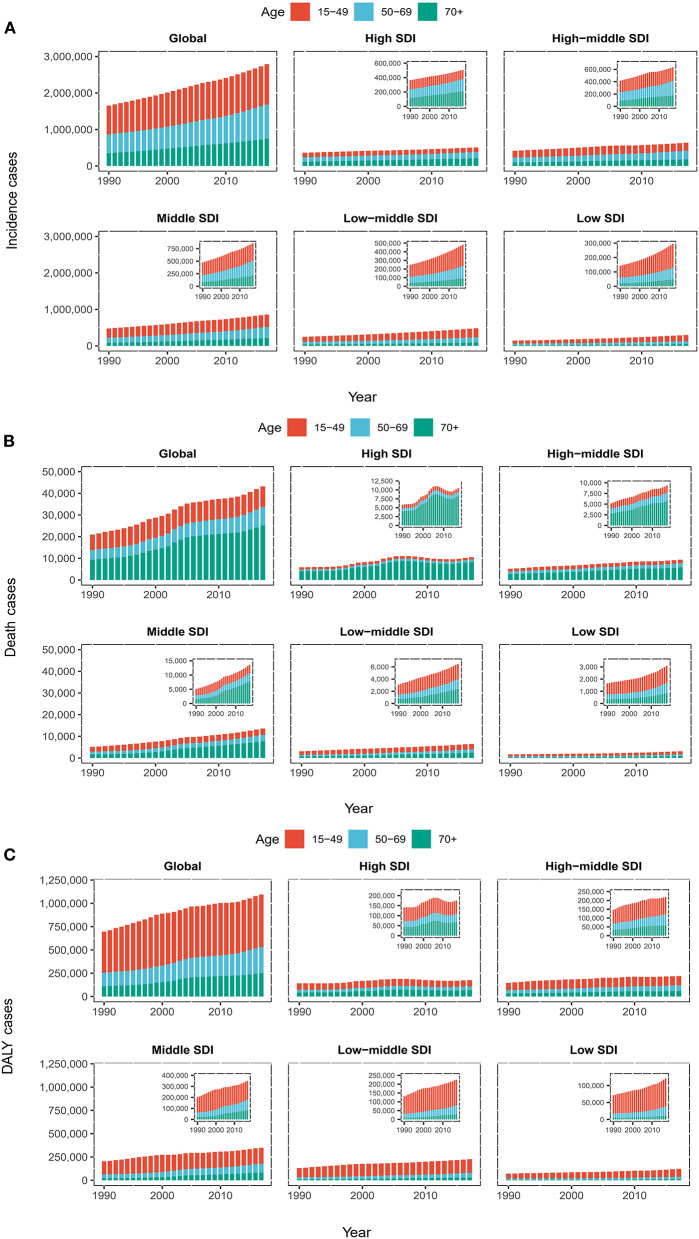
The proportion of the three age groups (15–49 years, 50–69 years and 70+ years) for myocarditis incidences **(A)**, deaths **(B)**, and DALY **(C)** cases globally and in five SDI quintiles between 1990 and 2017. SDI, Socio-Demographic Index; DALY, disability-adjusted life year.

**Figure 3:**
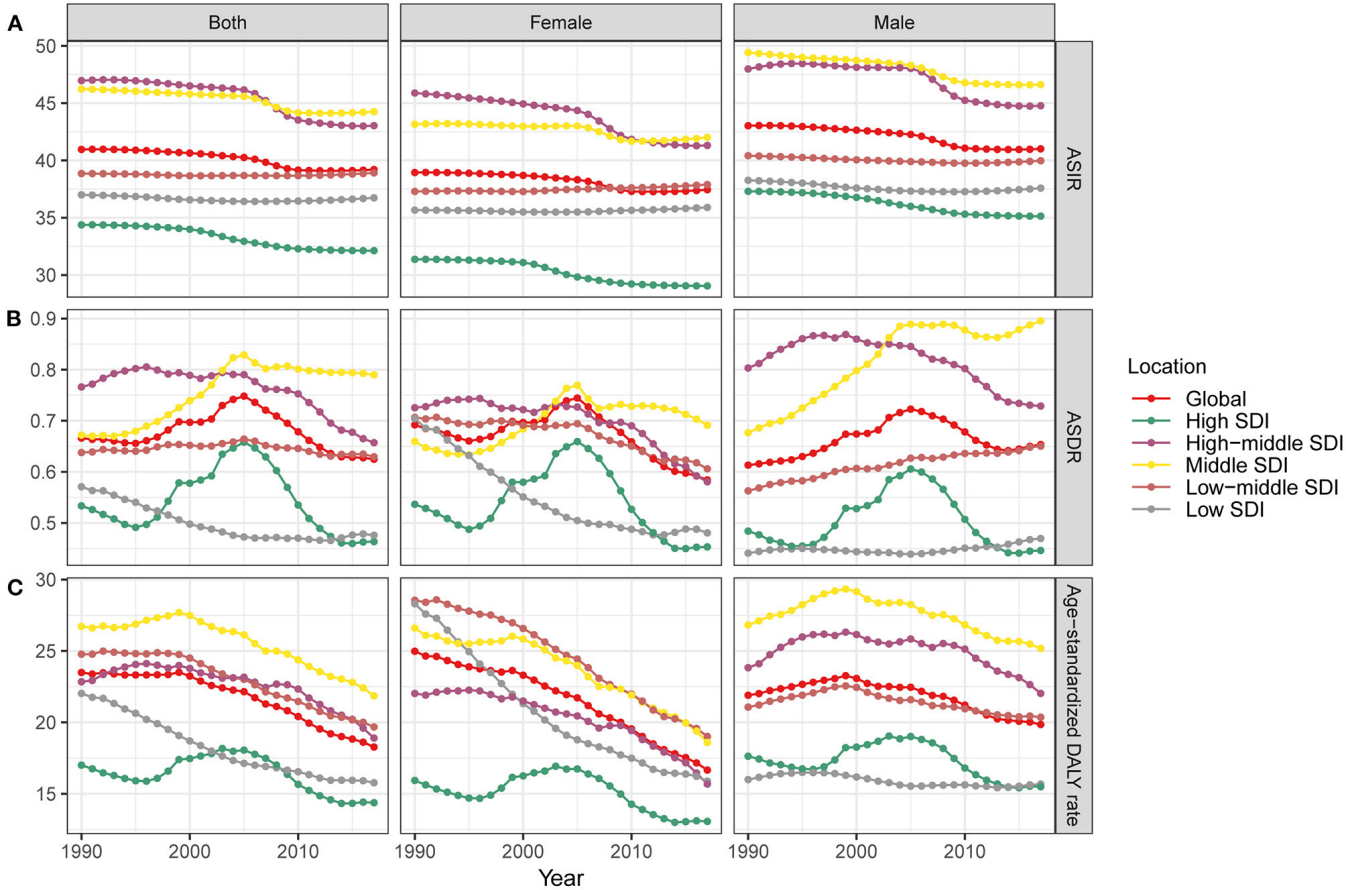
The trends of ASIR change **(A)**, ASDR **(B)**, and age-standardized DALY rate **(C)** (per 100,000 persons) globally and among different SDI quintiles between 1990 and 2017. ASIR, age-standardized incidence rate; ASDR, age-standardized death rate; DALY, disability-adjusted life year; SDI, Socio-Demographic Index.

The incidence of myocarditis increased across all the five SDI regions from 1990 to 2017, especially in the middle SDI (Table 1 and Figure 2A). The middle SDI quintile had the highest number of myocarditis incidences cases (945,000, 95%UI = 842,000–1,065,000) (Table 1). However, the ASIR decreased in the middle SDI, high-middle SDI, and high SDI regions with the EAPC of 0.0 (95% UI = −0.7 to 0.6), −0.3 (95% UI = −0.8 to −0.2) and −0.1 (95% UI = −1.2 to 1.0), respectively. The ASIR in the low SDI and low-middle SDI remained stable during the study period (Figure 3A and Table 1).

**Figure 4:**
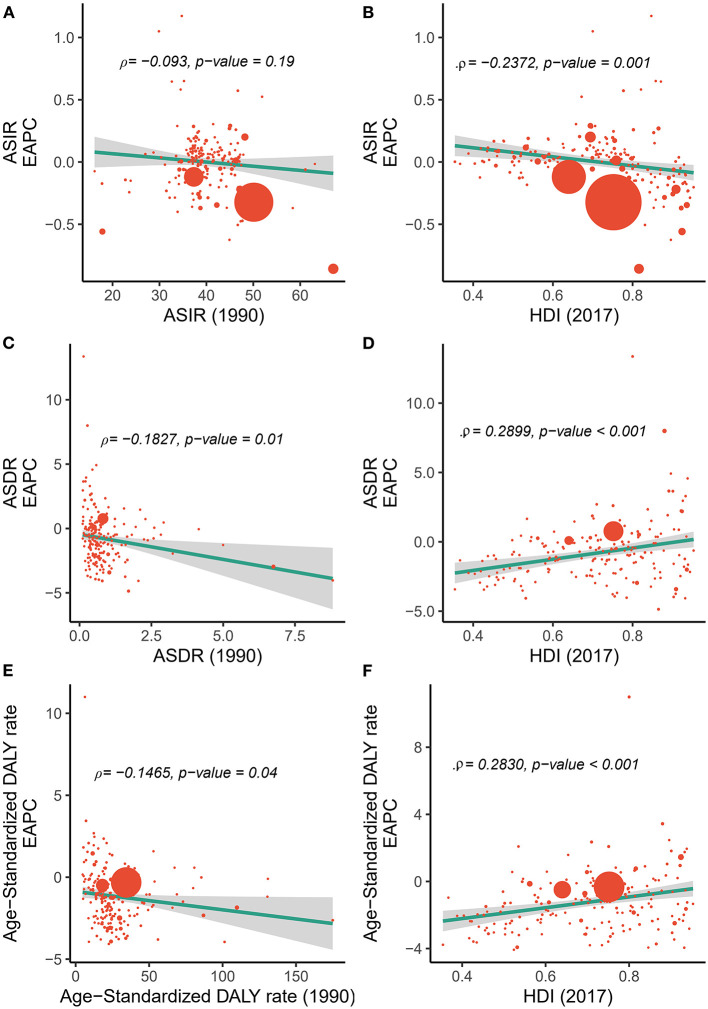
Correlation between the EAPC of incidences/deaths/DALYs and **(A,C,E)** the corresponding ASRs in 1990 and **(B,D,F)** HDI in 2017. The size of the circle is increased with the number of incidence, death, and DALY cases of myocarditis. ASR, age-standardized rate; ASIR, age-standardized incidence rate; ASDR, age-standardized death rate; DALY, disability-adjusted life year; EAPC, estimated annual percentage change; HDI, Human Development Index.

As shown in Figure 4, we found no significant correlation between the EAPC and the ASIR in 1990 (ρ = −0.093, *p* = 0.19, Figure 4A), and a negative correlation between the EAPC of ASIR and the HDI in 2017 (ρ = −0.24, *p* = 0.001, Figure 4B). These results illustrate that countries with a higher HDI have experienced a more rapid decrease in the ASIR of myocarditis from 1990 to 2017.

With respect to 21 GBD regions, the highest incidence in 2017 was observed in East Asia (792,000, 95% UI = 706,000–896,000), followed by South Asia (555,000, 95% UI = 494,000–628,000) (Table 1). The highest ASIR of 49.8/100,000 persons (95% UI = 44.51–55.82) was observed in Eastern Europe, and the lowest ASIR of 16.18/100,000 persons (95% UI = 14.86–17.68) was observed in high-income North America (Table 1). The greatest increase was observed in Western Sub-Saharan Africa (126.5%) followed by Central Sub-Saharan Africa (121.4%) (Table 1 and Supplementary Figure 1A). In 2017, Hungary, Montenegro, and Kyrgyzstan, had the highest myocarditis ASIRs for myocarditis, while Greenland and the United States were the top two countries with the lowest ASIRs (Supplementary Table 2 and Figures 1A,B).

#### Trends of Myocarditis Incidence Across Ages and Genders

Globally, the male and female genders experienced a similar increase in incidence from 1990 to 2017, while the ASIR of both genders remained relatively stable with an EAPC of −0.2 [95% confidence interval (CI) = −0.7–0.2], −0.1 (95% CI = −0.6–0.4), respectively (Table 1 and Figure 3A). Males achieved higher ASIR than females in five regions (Figures 3B,C). The incidence of myocarditis increased across all three age groups (15–49, 50–69, and 70+ years) from 1990 to 2017, and the 15–49 age group had the highest increase in incidence in all regions with low and high-middle SDIs. Regarding high SDI quintile, the 70+ years age group accounted for a larger proportion of the incidence (Figure 2A).

**Figure 5:**
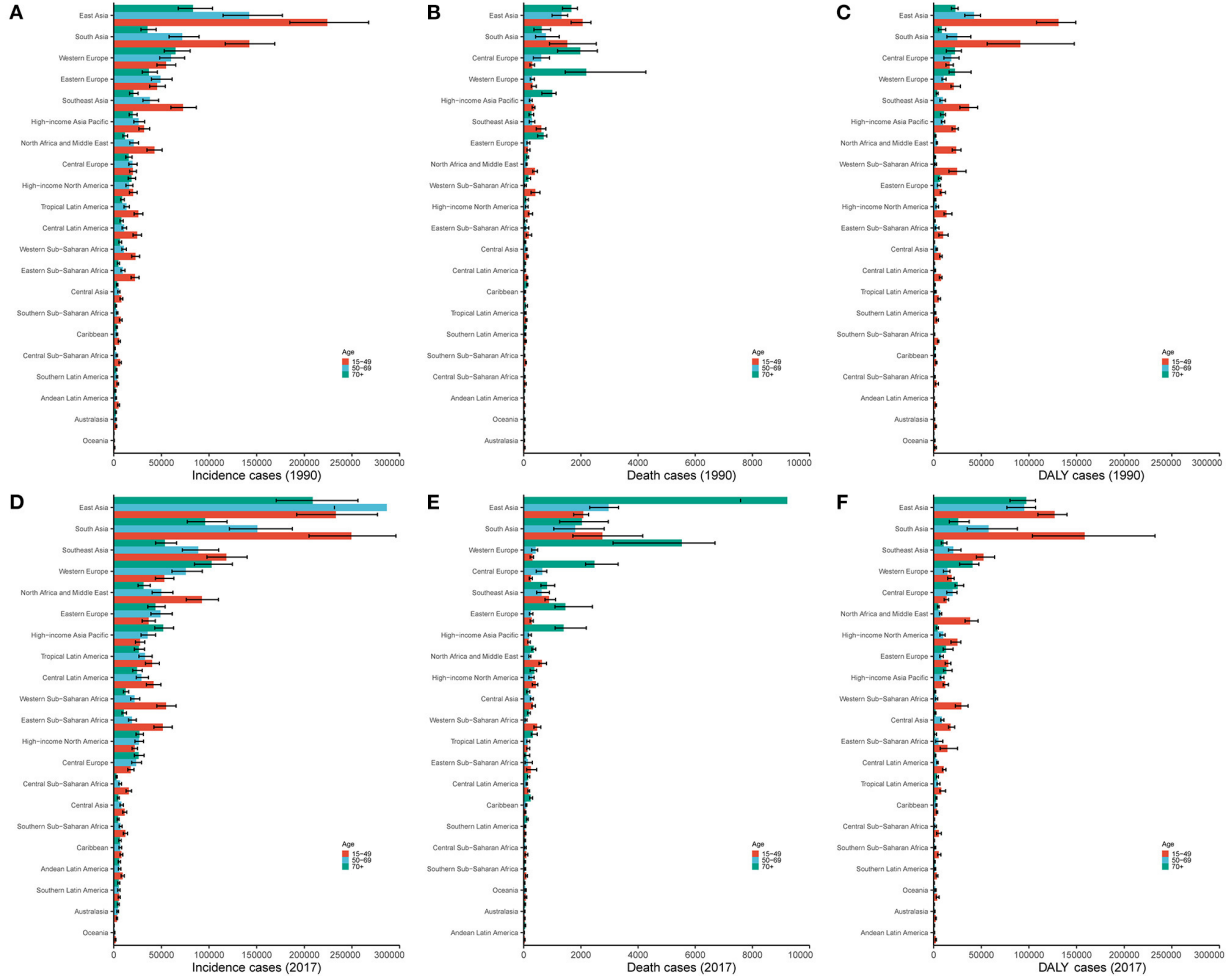
The proportion of the three age groups (15–49, 50–69, and 70+ years) for myocarditis incidence **(A,D)**, deaths **(B,E)**, and DALYs **(C,F)** in 21 GBD regions between 1990 and 2017. DALY, disability-adjusted life year; GBD, Global Burden of Disease.

**Figure 6:**
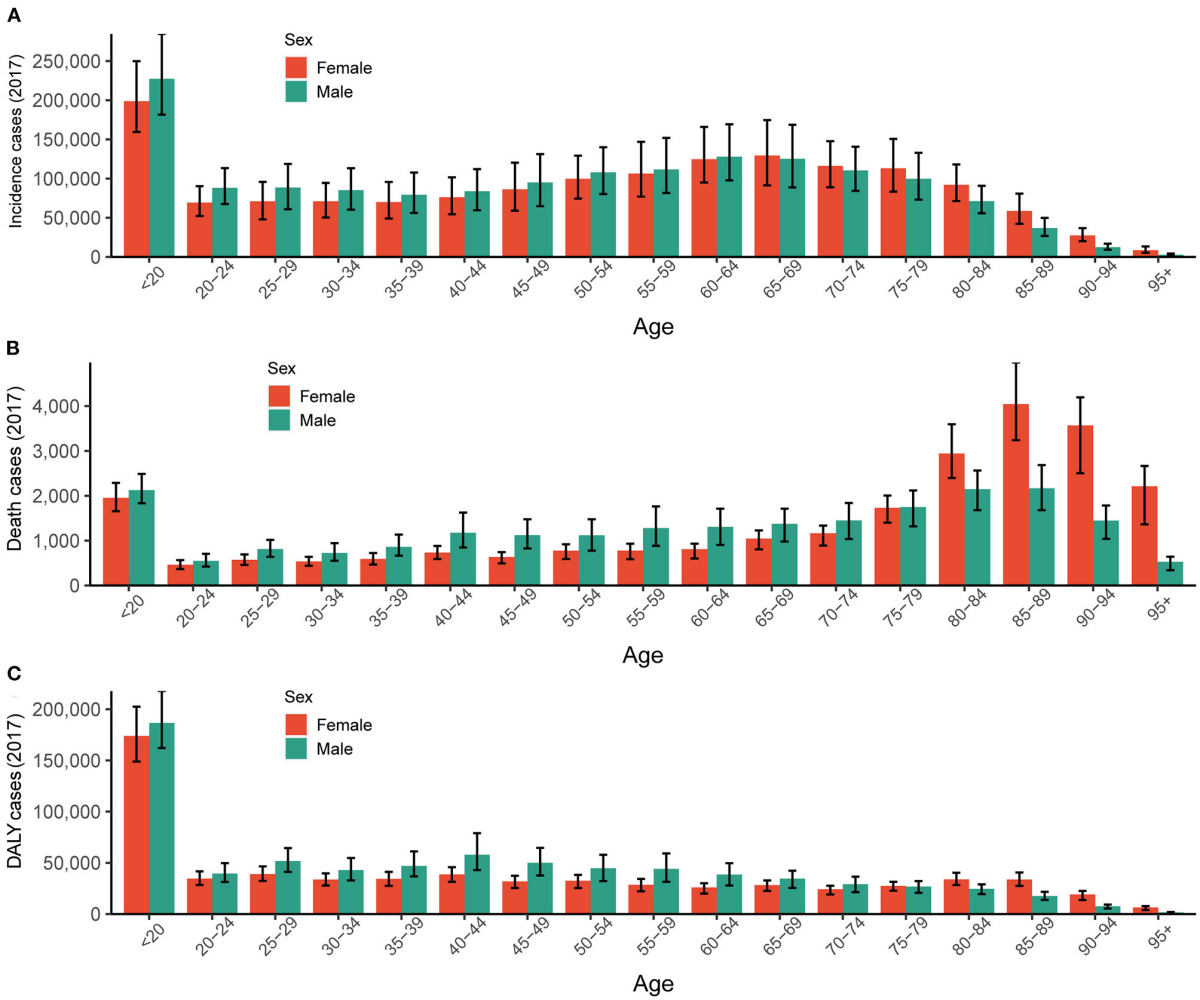
The global burden of myocarditis in both genders and different age groups in 2017. **(A)** The absolute number of incidence cases. **(B)** The absolute number of death cases. **(C)** The absolute number of DALYs. DALY, disability-adjusted life year.

Regionally, the incidence of myocarditis in East Asia and Western Europe grew rapidly, especially among elderly people (over 70 years old) (Figure 5A). In South Asia, the incidence of myocarditis in the young age group (15–49 years old) in 2017 increased sharply compared to that in 1990 (Figures 5A,D). Overall, the incidence of myocarditis varied largely between age groups and gender type, and the highest incidence was clearly observed in the group of people under the age of 20 for both males and females (Figure 6A).

### The Death Burden of Myocarditis

#### Trends of Myocarditis Death From 1990 to 2017

Globally, the annual death due to myocarditis increased gradually from 27,120 (95% UI = 21,810–110,574) in 1990 to 46,490 (95% UI = 39,710–51,820) in 2017. However, the global ASDR increased at the beginning of 1990 and then decreased over time (Table 1).

Regarding SDI level analysis, the number of myocarditis-related deaths increased across all five SDI quintiles from 1990 to 2017; it precipitously increased in the middle SDI (0.97-fold) and less obviously in the low SDI quintile (0.28-fold) (Table 2 and Figure 2B). Generally, the ASDR in the five SDI quintiles declined, and the middle SDI quintile had the highest ASDR (0.79, 95% UI = 0.64–0.79), which was higher than the world average (Table 2 and Figures 2B, 3B).

**Table 2:** The death cases and age-standardized death rate of myocarditis between 1990 and 2017 and its temporal trends from 1990 to 2017.

**Characteristics**	**1990**		**2017**		**1990–2017**
	**Death cases No. × 102 (95% UI)**	**ASDR per 100,000 No. (95% UI)**	**Death cases No. × 102 (95% UI)**	**ASDR per 100,000 No. (95% UI)**	**EAPC No. (95% CI)**
Global	271.2 (218.1–314.0)	0.7 (0.5–0.8)	464.9 (397.1–518.2)	0.6 (0.5–0.7)	−1.4 (−1.8 to −1.0)
Sex					
Female	153.1 (114.7–182.4)	0.7 (0.5–0.8)	245.6 (201.2–270.9)	0.6 (0.5–0.7)	−1.5 (−1.8 to −1.2)
Male	118.0 (93.7–138.9)	0.6 (0.5–0.7)	219.3 (178.3–265.9)	0.7 (0.5–0.8)	0.7 (0.4–1.1)
Andean Latin America	1.1 (0.8–1.4)	0.4 (0.3–0.5)	1.2 (1.0–1.4)	0.21 (0.18–0.24)	−2.5 (−2.8 to −2.1)
Australasia	0.9 (0.7–1.3)	0.4 (0.4–0.6)	1.2 (1.0–1.5)	0.31 (0.27–0.39)	−2.0 (−3.9–0.0)
Caribbean	2.7 (2.2–3.3)	1.0 (0.8–1.2)	4.2 (3.6–5.0)	0.84 (0.72–0.99)	−0.8 (−7.5–6.3)
Central Asia	3.0 (2.6–3.6)	0.6 (0.5–0.6)	7.9 (7.0–9.1)	1.01 (0.85–1.14)	−1.8 (−3.7–0.2)
Central Europe	29.6 (18.2–38.0)	2.4 (1.5–3.0)	20.1 (15.9–30.2)	0.56 (0.37–0.66)	−2.1 (−4.1 to −0.1)
Central Latin America	3.1 (2.5–3.5)	0.3 (0.2–0.3)	5.0 (4.5–6.2)	0.21 (0.15–0.26)	−0.3 (−3.4–2.8)
Central Sub-Saharan Africa	1.8 (1.1–2.7)	0.5 (0.3–0.7)	2.6 (1.5–4.2)	0.37 (0.20–0.59)	−0.6 (−1.7–0.6)
East Asia	74.6 (84.9)	0.8 (0.7–0.9)	153.7 (127.1)	1.0 (0.84–1.09)	−1.4 (−2.3 to −0.5)
Eastern Europe	10.3 (7.8–12.0)	0.5 (0.4–0.6)	20.1 (15.9–30.2)	0.64 (0.52–0.94)	0.1 (−1.1–1.3)
Eastern Sub-Saharan Africa	6.7 (4.2–10.9)	0.5 (0.3–0.8)	7.0 (3.4–12.7)	0.30 (0.12–0.60)	−1.0 (−5.5–3.7)
High-income Asia Pacific	16.8 (12.0–18.6)	1.0 (0.7–1.1)	17.8 (14.4–26.8)	0.39 (0.32–0.56)	−1.7 (−2.8 to −0.5)
High-income North America	5.1 (4.1–7.9)	0.2 (0.1–0.3)	12.4 (8.3–14.4)	0.29 (0.20–0.33)	−0.2 (−1.6–1.1)
North Africa and Middle East	14.7 (11.0–18.2)	0.5 (0.4–0.6)	16.9 (14.5–19.8)	0.34 (0.25–0.39)	−2.1 (−2.3 to −1.8)
Oceania	0.9 (0.7–1.4)	2.9 (2.2–4.0)	1.9 (1.4–2.6)	2.61 (1.98–3.39)	−0.6 (−1.8–0.6)
South Asia	37.5 (23.1–59.4)	0.5 (0.3–0.8)	70.3 (44.6–104.5)	0.55 (0.35–0.81)	1.0 (0.9–1.2)
Southeast Asia	17.3 (13.0–20.8)	0.5 (0.4–0.6)	26.1 (21.1–33.5)	0.47 (0.37–0.60)	−0.8 (−3.3–1.9)
Southern Latin America	2.2 (1.7–2.7)	0.5 (0.4–0.6)	2.5 (2.1–3.1)	0.32 (0.27–0.40)	−2.6 (−5.1–0.1)
Southern Sub-Saharan Africa	1.7 (1.5–2.1)	0.4 (0.4–0.5)	2.0 (1.6–3.0)	0.31 (0.25–0.47)	−3.4 (−6.6 to −0.1)
Tropical Latin America	3.6 (3.0–5.4)	0.3 (0.3–0.5)	6.6 (5.5–9.8)	0.31 (0.26–0.45)	−1.1 (−3.4–1.2)
Western Europe	28.6 (20.7–51.2)	0.5 (0.4–0.9)	62.9 (37.9–75.2)	0.56 (0.37–0.66)	−3.8 (−4.7 to −3.0)
Western Sub-Saharan Africa	8.6 (5.5–11.8)	0.8 (0.5–1.1)	8.7 (7.0–10.6)	0.4 (0.60–0.79)	−1.0 (−1.2 to −0.7)

We also found a significant negative relationship between the EAPC and the ASDR in1990 (ρ = −0.1827, *p* = 0.01, Pearson correlation analysis), suggesting that those countries with lower disease reservoirs at baseline experienced a more rapid decrease in ASDR (Figure 4C). A positive correlation was found between the EAPC of ASDR and the HDI in 2017 (ρ = 0.2899, *p* < 0.01, Pearson correlation analysis) (Figure 4D).

With respect to 21 GBD regions, the highest number of deaths in 2017 were observed in East Asia (15,370, 95% UI = 12,710–16,760), followed by South Asia (7,030, 95% UI = 4,460–10,450) (Table 2 and Supplementary Figure 2A). Regionally, the absolute number of myocarditis-related deaths increased in almost all GBD regions between 1990 and 2017, except for Central Europe, and the most pronounced increase was observed in East Asia (Supplementary Figure 1B). In 2017, China and India were found to have the highest reported number of myocarditis-related deaths, which may be a result of their larger population numbers or underlying risk factors. Bahrain and Latvia were the top two countries with the lowest number of myocarditis-related deaths (Supplementary Table 3, Supplementary Figures 2A,B).

#### Trends of Myocarditis Death Across Ages and Genders

Globally, deaths were more frequent among men (85%) than women (60%). The ASDR of males increased while the ASDR of females decreased from 1990 to 2017 (Table 2 and Figure 3). The myocarditis-related death cases increased across all three age groups (15–49, 50–69, and 70+ years) from 1990 to 2017, and the 70+ years age group had the highest number of deaths in all regions with low to high-middle SDIs (Figures 5B,E). Distinctly, the proportion of myocarditis-related deaths among elderly people was much larger in the high SDI, high-middle SDI, and middle SDI quintile, but in the low-middle SDI and low SDI quintile, the most pronounced number of deaths was observed in the young age group (15–49 years) (Figure 2B).

Regionally, the number of myocarditis-related deaths among the young age group (15–49 years old) in 2017 decreased compared to 1990. In contrast, the incidence of myocarditis-related deaths in East Asia and Western Europe grew rapidly, especially among elderly people (over 70 years old) (Figures 5B,E). Myocarditis-related deaths varied between age and sex groups. However, it is worth emphasizing that the group of people under the age of 20 for both males and females accounted for a large portion of overall number of deaths in 2017 (Figure 6B).

### The DALY Burden of Myocarditis

#### Trends of Myocarditis DALY From 1990 to 2017

Globally, the myocarditis-related DALY number in 2017 was 1,390,700 (95% UI = 1,224,200–1,470,400), and a 12.1% increase was noted for DALYs over the past 28 years. In contrast, the global age-standardized DALY rate decreased from 23.5/100,000 persons (95% UI = 19.3–27.7) in 1990 to 18.27/100,000 persons (95% UI = 16.1–20.4) in 2017, with an overall EAPC of −1.50 (95% UI = −2.30 to −0.8) (Table 3 and Figure 3C).

**Table 3:** The DALY and age-standardized DALY rate of myocarditis between 1990 and 2017 and its temporal trends from 1990 to 2017.

**Characteristics**	**1990**		**2017**		**1990–2017**
	**DALY no. × 10^**3**^ (95% UI)**	**Age-standardized DALY rate per 100,000 no. (95% UI)**	**DALY no. × 10^**3**^ (95% UI)**	**Age-standardized DALY rate per 100,000 no. (95% UI)**	**EAPC No. (95% CI)**
Global	1,240.2 (1,010.2–1,470.4)	23.5 (19.3–27.7)	1,390.7 (1,224.2–1,470.4)	18.27 (16.1–20.4)	−1.5 (−2.3 to −0.8)
Sex					
Female	670.5 (519.5–829.5)	25.0 (19.6–30.7)	646.1 (561.1–723.2)	16.7 (14.5–18.7)	−1.5 (−2.0 to −1.1)
Male	569.7 (437.9–670.6)	21.9 (17.5–25.7)	744.6 (624.8–893.8)	19.9 (16.8–23.7)	−0.3 (−0.6–0.0)
Andean Latin America	5.8 (4.2–7.2)	15.5 (11.4–19.0)	4.3 (3.7–5.2)	7.3 (6.3–8.6)	−2.8 (−3.3 to −2.3)
Australasia	4.1 (3.5–5.7)	20.9 (17.7–29.1)	4.5 (3.8–5.4)	14.9 (12.8–18.2)	−2.0 (−3.4 to −0.5)
Caribbean	10.6 (8.2–13.6)	31.3 (24.7–39.2)	11.4 (9.7–13.5)	24.4 (20.6–29.3)	−1.1 (−4.6–2.4)
Central Asia	14.1 (12.3–16.5)	22.2 (19.3–26.1)	31.2 (28.1–36.9)	34.7 (31.5–40.5)	−1.5 (−3.6–0.6)
Central Europe	64.4 (43.4–81.3)	49.1 (33.8–60.5)	60.4 (50.5–67.2)	35.4 (30.0–39.0)	−1.2 (−4.2–2.0)
Central Latin America	18.3 (14.2–20.5)	11.3 (8.9–12.6)	22.8 (20.5–27.4)	9.0 (8.1–10.9)	−0.2 (−2.9–2.5)
Central Sub-Saharan Africa	11.6 (6.9–17.9)	19.6 (12.3–28.2)	14.3 (8.7–24.4)	13.3 (8.1–20.8)	−1.3 (−2.0 to −0.6)
East Asia	408.2 (327.9–472.1)	33.9 (27.4–39.0)	415.8 (353.9–451.3)	29.2 (25.2–31.5)	−2.9 (−4.1 to −1.8)
Eastern Europe	25.1 (20.9–31.3)	11.2 (9.3–13.7)	38.6 (32.6–48.9)	15.2 (13.0–18.5)	0.4 (−2.7–3.7)
Eastern Sub-Saharan Africa	44.2 (27.6–70.9)	20.3 (12.9–31.9)	40.2 (22.7–68.2)	11.7 (6.0–20.3)	−1.0 (−5.2–3.4)
High-income Asia Pacific	51.0 (40.9–56.6)	29.3 (22.9–32.5)	36.0 (29.8–46.7)	14.5 (12.3–18.1)	−1.7 (−2.6 to −0.8)
High-income North America	29.5 (24.0–42.2)	11.2 (9.2–16.2)	51.5 (37.6–58.9)	15.5 (11.5–17.5)	−0.7 (−1.6–0.3)
North Africa and Middle East	102.5 (72.3–128.0)	25.4 (19.0–31.2)	93.4 (79.4–110.3)	15.9 (13.5–18.6)	−2.4 (−3.6 to −1.2)
Oceania	4.3 (3.1–6.3)	85.9 (63.8–125.6)	8.1 (6.0–11.2)	78.8 (59.7–106.1)	−0.7 (−3.4–2.0)
South Asia	196.7 (130.0–306.4)	19.2 (12.2–29.9)	282.3 (190.2–401.1)	17.3 (11.6–24.7)	0.1 (−0.4–0.6)
Southeast Asia	99.6 (74.4–126.1)	21.8 (16.7–26.4)	109.7 (94.8–132.3)	17.4 (15.0–20.8)	−0.9 (−2.8–0.9)
Southern Latin America	9.3 (7.5–11.3)	19.2 (15.4–23.2)	7.6 (6.4–9.7)	11.1 (9.3–14.6)	−2.8 (−5.1 to −0.3)
Southern Sub-Saharan Africa	9.6 (8.2–11.3)	18.7 (16.3–22.0)	10.2 (7.8–15.4)	13.5 (10.4–20.1)	−3.6 (−6.8 to −0.3)
Tropical Latin America	21.3 (17.6–30.6)	14.0 (11.8–20.2)	22.5 (18.6–32.7)	11.0 (9.0–15.9)	−1.5 (−3.8–1.0)
Western Europe	61.1 (51.4–87.9)	13.9 (11.8–19.3)	78.5 (59.0–89.4)	12.0 (9.2–13.6)	−2.7 (−3.8 to −1.6)
Western Sub-Saharan Africa	48.7 (30.8–67.0)	25.3 (16.4–34.3)	47.4 (38.3–56.9)	12.4 (10.1–14.8)	−1.0 (−2.3–0.4)

The global myocarditis-related DALY number in 2017 was 1,390,700 (95% UI = 1,224,200–1,470,400), while the middle SDI and low-middle SDI quintile accounted for the majority of the DALY number (Table 3 and Figure 2C). The middle SDI quintile had the highest age-standardized DALY rate (21.9/100,000 persons, 95% UI = 18.8–25.4), while the high SDI quintile had the lowest age-standardized DALY rate (14.4/100,000 persons, 95% UI = 12.2–15.7) (Table 3 and Figure 3C). The age-standardized DALY rate declined most seriously in the high-middle SDI quintile with an EAPC of −2.0 (95% UI = −3.7 to −0.4) (Table 3 and Figure 3C).

The results also revealed a significant negative relationship between EAPC and the age-standardized DALY rate in 1990 (ρ = −0.1465, *p* = 0.04) (Figure 4E), contrary to a positive pattern of EAPC of DALYs and the HDI in 2017 (ρ = 0.2830, *p* < 0.01) (Figure 4F).

With respect to 21 GBD regions, the highest DALY number in 2017 was observed in East Asia (415,800, 95% UI = 353,900–451,300), followed by South Asia (282,300, 95% UI = 190,200–401,100) (Table 3). South Asia displayed the largest increase in myocarditis DALY numbers between 1990 and 2017 (Table 3). However, the myocarditis DALY number in the high-income Asia Pacific region and Southern Latin America decreased from 1990 to 2017 (Table 3 and Figures 5C,F).

#### Trends of Myocarditis DALY Across Ages and Genders

Globally, the age-standardized DALY rate decreased for both genders, while a more pronounced decline was noted for women from 1990 to 2017 (Table 3 and Figure 3C). The proportion of the three age groups (15–49, 50–69, and 70+ years) in myocarditis DALY number remained relatively stable between 1990 and 2017. Myocarditis-related DALY number in the 15–49 years age group remained the highest across all of the SDI quintiles (Figure 2C).

The age-standardized DALY rate in women was higher than that in men subjects in 1990, while in 2017, the age-standardized DALY rate was higher in men than women (Table 3 and Figure 3C). The myocarditis DALY number at a young age in 2017 (15–49 years old group) remained stable when compared to the data in 1990. In East Asia, the 15–49 years group accounted for most of the DALY number in 1990, while in 2017, the 70+ years group experienced a sharp increase compared to 1990. In South Asia, people aged 15–49 years accounted for the largest proportion (Figures 5C,F). Overall, the myocarditis DALY cases varied across different age groups and genders, and the largest number of DALY was clearly observed in the group of people under the age of 20 for both males and females (Figure 6C).

## Discussion

The current study systematically evaluated the global epidemiological data of myocarditis and demonstrated that myocarditis has been confirmed as a constantly developing condition due to its progressive and significant effects on mortality and disability. Some specific differences were identified based on age, gender, nation, SDI quintiles, and GBD regions, and these heterogeneities were likely to have significant implications for global public health and health policymaking related to myocarditis.

In general, myocarditis increased in incidence, death, and DALY cases, while the global ASR for these three metrics decreased between 1990 and 2017. Population growth and aging in many regions increased the myocarditis disease burden worldwide (22, 23), and the change in diagnostic method from clinical ± bx to MRI (non-invasive) may have also led to an increase in the incidence, mortality, and DALY cases of myocarditis. Regarding the different age stratifications and SDI quintiles, the group under the age of 20 had a higher myocarditis burden compared with other age groups, and middle SDI quintiles had higher ASR for the incidence, death, and DALY. Another finding was that the ASDR trend for myocarditis has plateaued since 2007 and has shown no decline in middle SDI quintile, indicating that a targeted control and prevention strategy should be developed to reduce the myocarditis burden in these regions.

Understanding the temporal trends of the myocarditis disease burden facilitates the initiation of more targeted public health strategies. Although a 59.6% increase in incidence cases, 71.4% increase in deaths cases, and 12.1% increase in DALY cases were observed over the past 28 years across the world, the global ASIR, ASDR, and age-standardized DALY rate showed a declining trend with negative EAPC values. As for the analysis of SDI levels, the incidence, death, and DALY cases of myocarditis increased in all SDI quintiles between 1990 and 2017, increasing sharply in the middle SDI and high-middle SDI quintiles and gently increasing in low SDI quintile. Correspondingly, the ASIR, ASDR, and age-standardized DALY rates in five SDI quintiles declined or remained stable during the same period. From a regional perspective, the most pronounced increase in myocarditis disease burden was observed in Virgin Islands, United States, Czech Republic, Andorra, Qatar, and Uzbekistan, while Libya, Poland, Singapore, Austria, and Rwanda had made substantial strides in decreasing incidence numbers. Our study adds to the accumulating evidence that myocarditis is another component of the recognized epidemiological transition. This phenomenon must be considered for policymakers to allocate limited resources and formulate relevant policies more rationally.

From 1990 to 2017, the global ASIR trend of myocarditis varied considerably between men and women, and the ASIR in males was higher than that in females during the same period, both globally and in the five SDI quintiles. In addition to the higher incidence of myocarditis in males (24, 25), previous studies also demonstrated that males have a higher risk of myocarditis compared to females (26–28). Some clinical trials also displayed a slight male preponderance in the incidence of myocarditis, with the women/men ratio ranging from 1:1.5 to 1:1.7 (24, 25). Little is known about the mechanism of this apparent male preponderance in myocarditis, but some studies have shown that sex hormones and additional genetic variations may be responsible for the differences in left ventricular recovery, epidemiology, and survival between men and women (29). With respect to age, elderly people may be more likely to be infected by myocarditis due to frailty (30).

In 2017, the incidence and death cases displayed a bimodal distribution as age increased, and the population under the age of 20 accounted for a large proportion of incidence, death, and DALY cases. People aged 65–69 and 85–89 years also had high rates of incidence and death, respectively. These data demonstrated that younger people and older people accounted for a major global burden of myocarditis in 2017. Consistent with our data, previous reports demonstrated that both the incidence of myocarditis and sudden cardiovascular death (SCD) attributed to myocarditis in children and young adults were significantly higher than those in middle-aged and older adults (26, 31, 32). As for the different ages and genders, Kytö et al. found that myocarditis occurred in all ages, and young men were more susceptible compared to their age-matched female counterparts. The distribution of myocarditis in female patients was significantly more stable across all age groups and showed no increase in young adults (32). However, our data showed that people under the age of 20 years had the highest incidence, number of deaths, and DALY cases both in men and women. Testosterone may account for the development of myocarditis in different ages and genders. Previous experimental data showed that increased virus binding to myocytes (33), commitment to T helper type 1 immune response (34), inhibition of anti-inflammatory cell populations (35), and upregulation of cardiac fibrotic remodeling genes (29) were the possible mechanisms of testosterone action in myocarditis.

Moreover, our results indicated a significantly negative association between the EAPC of ASR and the baseline ASR (in 1990), and a significantly positive association between the EAPC of ASR and HDI (in 2017) for both death and DALY. The ASIR of myocarditis in 1990 reflected the disease reservoir at baseline, and the HDI in 2017 can serve as a surrogate for the level and availability of health care in each country. The results could be explained by the fact that countries with higher ASDR and age-standardized DALY rates were more likely to consider myocarditis as a priority in prevention programs in terms of public health considerations. The decline in ASIR was more pronounced in countries with high HDI, possibly because of the better healthcare systems in these countries.

To allocate limited resources more reasonably in the health care system and evaluate the impact of myocarditis control and benchmark progress in their nations, policymakers need the country-specific information about myocarditis disease burden. Considering the fact that most of the existing data are of low quality, sparse, or with limited scope, the GBD study aimed to provide up-to-data estimates of disease incidence, mortality, and DALY, which can be used by stakeholders to gain knowledge of the trend of myocarditis in their local area.

Although the GBD study can be used to fill the remaining knowledge gap, there are still several limitations. First, the quality of the globally included information might be confounded by a multitude of factors, such as the differences in data collecting and the quality of data sources, which remains inevitable in this type of analysis using the ICD-10 codes. Second, the classifications of myocarditis are not available in this study, and it is well-known that the clinical course varies depending on the type of myocarditis. This should be investigated in future studies. Third, the identification of myocarditis has improved remarkably over the last decade with the introduction of high-sensitivity troponins and cardiac magnetic resonance imaging. However, it is difficult to measure the influence of diagnostic methods, and further studies are needed to confirm these results.

## Conclusion

In general, there was a huge increase in total cases of myocarditis and a slight decrease in ASR in terms of the incidence, death, and DALY, worldwide between 1990 and 2017. The ASIR, ASDR, and age-standardized DALY rate in the middle SDI quintile were higher than in the low and high SDI regions, suggesting that a larger investment was needed to reduce the myocarditis burden in middle SDI regions. Notably, this changing pattern was also heterogeneous among the gender and age groups. The total myocarditis burden was higher in the group of people under the age of 20, and the cases and ASR of the incidence, death, and DALY in the male population were always higher than those in the female population, globally or regionally. Increased awareness regarding myocarditis and timely treatment are important for these young people in clinical practice. These results highlight the need for policies to strengthen the screening, prevention, and treatment of myocarditis in children and young adults, especially in men. The heterogeneities of myocarditis disease burden illustrated by our study should be considered by policymakers to rationalize limited resources and formulate relevant policies.

## Data Availability Statement

The original contributions presented in the study are included in the article/Supplementary Material, further inquiries can be directed to the corresponding author.

## Author Contributions

XW conceived the study, analyzed the data, and wrote the manuscript. XB analyzed the data and revised the manuscript. LW, JL, and DY revised the manuscript and reviewed the results. DM and AM revised the manuscript and provided comments of this research. TH revised the manuscript and provided guidance for this study. All authors contributed to the article and approved the submitted version.

## Conflict of Interest

The authors declare that the research was conducted in the absence of any commercial or financial relationships that could be construed as a potential conflict of interest.

## Supplementary Material

Supplementary Figure 1 The global disease burden of myocarditis for both genders in 195 countries and territories. **(A)** The relative change in incident cases of myocarditis between 1990 and 2017. **(B)** The relative change in myocarditis-related deaths between 1990 and 2017. **(C)** The relative change in DALY cases of myocarditis between 1990 and 2017. ASIR, age-standardized incidence rate; ASDR, age-standardized death rate; DALY, disability-adjusted life year; EAPC, estimated annual percentage change.

Supplementary Figure 2 The global death burden of myocarditis in 195 countries and territories. **(A)** The absolute number of myocarditis incidence cases in 2017. **(B)** The ASDR (per 100,000 persons) of myocarditis in 2017. **(C)** The EAPC of myocarditis ASDRs between 1990 and 2017. ASDR, age-standardized death rate; EAPC, estimated annual percentage change.

Supplementary Figure 3 The global DALY burden of myocarditis in 195 countries and territories. **(A)** The absolute number of myocarditis DALY cases in 2017. **(B)** The age-standard DALY rate (per 100,000 persons) of myocarditis in 2017. **(C)** The EAPC of myocarditis age-standardized DALY rate between 1990 and 2017. DALY, disability-adjusted life year; EAPC, estimated annual percentage change.
