# Supplementary material for: Global, Regional, and National Burden of Myocarditis From 1990 to 2017: A Systematic Analysis Based on the Global Burden of Disease Study 2017
Source: Front Cardiovasc Med. 2021 Jul 2;8:692990. doi: 10.3389/fcvm.2021.692990 (PMC8284556; doi:10.3389/fcvm.2021.692990)
Supplement: Supplementary file 1 [file Table_1.docx]

**Table S1** ICD-10 code and subgroups of GBD cause code B.2.6.1 (Myocarditis)

| Code | Disease |
| --- | --- |
| I40 | **Acute myocarditis** |
| I40.0 | Infective myocarditis |
| I40.1 | Isolated myocarditis |
| I40.8 | Other acute myocarditis |
| I40.9 | Acute myocarditis, unspecified |
| I41 | **Myocarditis in diseases classified** |
| I41.0 | Myocarditis in bacterial diseases |
| I41.1 | Myocarditis in viral diseases |
| I41.2 | Myocarditis in other infectious and parasitic diseases |
| I41.8 | Myocarditis in other diseases |
| I51.4 | **Myocarditis, unspecified** |

GBD, Global Burden of Disease; ICD-10, International Classification of Disease 10.
