# Supplementary material for: Global, Regional, and National Burden of Myocarditis From 1990 to 2017: A Systematic Analysis Based on the Global Burden of Disease Study 2017
Source: Front Cardiovasc Med. 2021 Jul 2;8:692990. doi: 10.3389/fcvm.2021.692990 (PMC8284556; doi:10.3389/fcvm.2021.692990)
Supplement: Supplementary file 2 [file Table_2.docx]

**Table S2** The incidence cases and age-standardized incidence rate of myocarditis between 1990 and 2017, and its temporal trends from 1990 to 2017 in 195 countries and territories.

|  | 1990 |  | 2017 |  | 1990 to 2017 |
| --- | --- | --- | --- | --- | --- |
| Countries and territories | Incidence cases No. (95% UI) | ASIR per 100,000 No. (95% UI) | Incident cases No. (95% UI) | ASIR per 100,000 No. (95% UI) | EAPC No. (95% CI) |
| Afghanistan | 3269.8(2895.3-3708.4) | 41.3(36.8-46.8) | 8343.7(7331.6-9578.6) | 41.1(36.5-46.3) | 0.00(-0.02-0.02) |
| Albania | 981.6(876.0-1096.4) | 35.7(31.8-39.8) | 1198.3(1065.5-1357.0) | 34.8(31.0-39.1) | -0.09(-0.09-0.09) |
| Algeria | 6664.4(5912.5-7556.3) | 37.0(32.8-41.8) | 14067.4(12458.2-15855.6) | 38.2(34.1-43.1) | 0.11(0.100.10) |
| American Samoa | 17.0(15.1-19.1) | 47.9(42.8-53.7) | 23.6(21.1-26.7) | 48.8(43.7-54.8) | 0.06(0.040.04) |
| Andorra | 21.6(19.0-24.6) | 37.2(33.3-42.2) | 38.0(33.7-43.2) | 35.3(31.6-39.9) | -0.30(-0.35-0.35) |
| Angola | 2526.4(2221.2-2858.1) | 38.8(34.5-43.7) | 7042.7(6171.1-8018.7) | 39.3(35.0-44.3) | -0.01(-0.03-0.03) |
| Antigua and Barbuda | 23.2(20.7-26.1) | 41.1(36.7-46.5) | 40.7(36.3-46.3) | 42.1(37.6-47.7) | 0.10(0.090.09) |
| Argentina | 7411.2(6580.3-8384.2) | 22.7(20.2-25.6) | 11111.9(9870.9-12557.8) | 22.5(20.0-25.4) | -0.14(-0.20-0.20) |
| Armenia | 1124.3(996.4-1269.2) | 37.1(33.0-41.7) | 1237.9(1100.5-1399.3) | 34.4(30.7-38.6) | -0.25(-0.29-0.29) |
| Australia | 5867.4(5250.8-6670.0) | 31.5(28.2-35.7) | 9908.4(8828.1-11215.8) | 30.6(27.4-34.4) | -0.23(-0.28-0.28) |
| Austria | 4647.7(4136.1-5240.5) | 47.0(42.1-52.7) | 6073.1(5429.1-6868.3) | 46.9(42.1-52.4) | 0.00(-0.01-0.01) |
| Azerbaijan | 2077.2(1845.6-2334.5) | 33.9(30.4-38.1) | 3008.6(2667.6-3392.2) | 31.1(27.7-35.0) | -0.40(-0.45-0.45) |
| Bahrain | 143.8(124.7-164.4) | 40.2(35.9-45.3) | 514.3(443.5-590.3) | 39.6(35.1-45.0) | -0.10(-0.12-0.12) |
| Bangladesh | 25216.2(22188.3-28449.7) | 34.2(30.5-38.6) | 46841.3(41543.3-53063.9) | 33.6(30.0-38.1) | -0.06(-0.09-0.09) |
| Barbados | 127.5(113.5-144.3) | 45.3(40.4-50.9) | 177.9(158.1-202.6) | 45.2(40.4-51.3) | -0.02(-0.03-0.03) |
| Belarus | 4245.7(3779.4-4813.6) | 36.1(32.3-40.7) | 4390.4(3894.7-4998.3) | 34.9(31.2-39.4) | -0.15(-0.17-0.17) |
| Belgium | 3891.9(3451.5-4412.0) | 30.4(27.1-34.1) | 5141.6(4551.5-5856.2) | 30.9(27.7-34.7) | 0.04(0.020.02) |
| Belize | 55.6(49.4-62.6) | 41.3(36.9-46.5) | 138.0(122.6-155.3) | 42.2(37.8-47.5) | 0.06(0.050.05) |
| Benin | 1163.6(1024.4-1313.7) | 37.1(32.9-42.0) | 2848.6(2521.6-3229.8) | 37.3(33.2-42.4) | 0.01(0.000.00) |
| Bermuda | 27.6(24.4-31.3) | 43.8(39.1-49.4) | 41.9(37.2-47.8) | 43.7(38.9-49.4) | -0.05(-0.09-0.09) |
| Bhutan | 131.9(115.8-150.0) | 34.1(30.3-38.6) | 271.4(238.2-306.7) | 34.0(30.3-38.3) | 0.00(-0.03-0.03) |
| Bolivia | 1716.7(1527.5-1945.9) | 37.6(33.7-42.6) | 3862.8(3431.3-4363.9) | 39.0(34.7-44.1) | 0.13(0.120.12) |
| Bosnia and Herzegovina | 1897.5(1685.2-2140.6) | 44.2(39.6-49.7) | 1910.1(1696.0-2166.6) | 41.9(37.4-47.0) | -0.20(-0.22-0.22) |
| Botswana | 361.1(318.2-408.8) | 40.3(35.8-45.3) | 756.2(668.1-853.5) | 41.2(36.6-46.4) | 0.06(0.050.05) |
| Brazil | 54520.4(48574.3-61320.7) | 46.3(41.5-52.1) | 103879.4(92495.6-117294.5) | 46.7(41.8-52.6) | 0.01(0.000.00) |
| Brunei | 86.3(75.7-98.2) | 44.3(39.7-49.8) | 158.8(140.5-179.6) | 40.9(36.7-45.9) | -0.37(-0.39-0.39) |
| Bulgaria | 4015.5(3551.3-4550.3) | 37.7(33.7-42.3) | 3887.0(3427.6-4436.8) | 36.4(32.4-40.9) | -0.10(-0.13-0.13) |
| Burkina Faso | 2380.3(2114.5-2709.1) | 37.7(33.7-42.8) | 5268.6(4649.2-5946.7) | 37.9(33.7-43.0) | 0.01(0.000.00) |
| Burundi | 1402.6(1235.6-1596.5) | 39.9(35.4-45.0) | 2755.6(2416.6-3126.9) | 39.4(35.0-44.2) | -0.12(-0.15-0.15) |
| Cambodia | 3196.4(2822.6-3602.6) | 43.9(39.0-49.5) | 6068.5(5381.2-6881.8) | 44.6(39.9-50.5) | 0.05(0.040.04) |
| Cameroon | 2536.4(2244.9-2862.6) | 37.1(33.1-41.9) | 7048.9(6193.0-8023.5) | 37.9(33.6-43.0) | 0.08(0.060.06) |
| Canada | 5466.1(4837.9-6192.9) | 17.8(15.8-20.1) | 8751.8(7745.1-9979.2) | 17.1(15.2-19.3) | -0.17(-0.21-0.21) |
| Cape Verde | 107.2(95.4-121.1) | 38.8(34.6-43.8) | 196.4(174.5-220.8) | 39.6(35.2-44.7) | 0.10(0.080.08) |
| Central African Republic | 683.7(601.5-772.2) | 38.2(34.1-43.4) | 1227.2(1079.7-1398.7) | 38.4(34.3-43.4) | -0.01(-0.04-0.04) |
| Chad | 1511.3(1337.1-1701.3) | 37.1(33.0-41.9) | 3470.8(3039.4-3932.1) | 37.2(33.1-42.1) | -0.01(-0.02-0.02) |
| Chile | 2758.3(2452.1-3106.3) | 23.5(21.0-26.6) | 4773.9(4227.8-5427.6) | 22.8(20.2-25.7) | -0.24(-0.29-0.29) |
| China | 514831.4(457721.6-576046.1) | 50.1(44.9-56.2) | 755736.5(673097.5-855098.9) | 46.9(42.0-52.6) | -0.32(-0.38-0.38) |
| Colombia | 10265.6(9116.5-11580.2) | 41.5(37.0-46.9) | 22142.2(19691.9-24979.0) | 41.9(37.3-47.3) | 0.01(0.000.00) |
| Comoros | 117.0(103.2-133.0) | 38.1(33.9-43.2) | 219.6(193.6-248.0) | 38.5(34.2-43.4) | -0.03(-0.05-0.05) |
| Congo | 655.7(577.1-740.0) | 40.2(35.8-45.3) | 1422.6(1248.4-1602.4) | 39.8(35.6-45.0) | -0.07(-0.10-0.10) |
| Costa Rica | 1027.8(910.6-1153.8) | 43.4(38.5-48.7) | 2144.0(1900.1-2427.1) | 44.0(39.1-49.8) | 0.03(0.010.01) |
| Cote d'Ivoire | 2964.6(2601.0-3378.7) | 39.3(35.1-44.2) | 6524.5(5725.3-7437.9) | 38.9(34.6-44.1) | -0.08(-0.10-0.10) |
| Croatia | 2624.4(2343.0-2967.4) | 46.7(41.9-52.6) | 2627.2(2332.5-2988.9) | 41.4(37.1-46.3) | -0.51(-0.58-0.58) |
| Cuba | 5074.0(4519.6-5748.2) | 46.7(41.7-52.7) | 8008.7(7111.1-9068.4) | 52.6(46.9-59.0) | 0.57(0.510.51) |
| Cyprus | 302.6(268.5-341.1) | 37.2(33.3-41.9) | 583.7(520.0-661.4) | 36.8(32.8-41.4) | -0.10(-0.12-0.12) |
| Czech Republic | 4879.4(4346.3-5515.3) | 40.8(36.5-46.0) | 5769.0(5125.9-6560.4) | 38.2(34.2-42.8) | -0.18(-0.24-0.24) |
| Democratic Republic of the Congo | 9464.3(8353.3-10764.1) | 38.2(34.1-43.2) | 20542.9(18025.2-23262.8) | 38.1(33.8-43.0) | -0.03(-0.04-0.04) |
| Denmark | 2083.3(1860.2-2360.1) | 31.2(27.9-35.2) | 2590.6(2277.7-2951.0) | 31.1(27.8-35.1) | -0.02(-0.07-0.07) |
| Djibouti | 110.8(96.1-127.1) | 36.6(32.5-41.2) | 315.7(278.1-357.5) | 38.5(34.5-43.3) | 0.17(0.160.16) |
| Dominica | 31.8(28.3-36.1) | 43.8(39.1-49.7) | 36.3(32.4-40.8) | 44.2(39.6-49.7) | 0.04(0.030.03) |
| Dominican Republic | 2124.0(1879.4-2403.7) | 39.6(35.2-44.8) | 4094.7(3655.0-4600.2) | 41.4(37.0-46.7) | 0.15(0.140.14) |
| Ecuador | 2879.7(2558.1-3255.4) | 38.4(34.3-43.4) | 6378.7(5673.5-7204.5) | 40.5(36.1-45.8) | 0.25(0.230.23) |
| Egypt | 14727.2(12969.3-16828.2) | 37.3(33.2-42.1) | 30042.6(26455.1-33964.9) | 39.8(35.4-44.8) | 0.29(0.270.27) |
| El Salvador | 1553.1(1374.1-1753.0) | 39.0(34.6-44.2) | 2360.2(2109.5-2665.3) | 39.7(35.4-44.8) | 0.05(0.050.05) |
| Equatorial Guinea | 110.0(96.6-124.5) | 39.0(34.8-44.2) | 346.2(301.1-393.7) | 39.6(35.3-44.7) | 0.01(-0.01-0.01) |
| Eritrea | 642.0(558.5-737.9) | 36.8(32.7-41.6) | 1438.7(1258.1-1648.9) | 37.0(32.9-42.0) | 0.02(-0.01-0.01) |
| Estonia | 592.2(528.8-666.4) | 32.6(29.2-36.5) | 709.9(628.9-807.8) | 36.6(32.7-41.3) | 0.65(0.490.49) |
| Ethiopia | 12130.8(10648.1-13838.9) | 37.7(33.4-42.8) | 26043.2(22919.3-29701.2) | 38.8(34.4-43.9) | 0.09(0.080.08) |
| Federated States of Micronesia | 32.0(28.2-36.4) | 42.8(38.2-48.3) | 37.7(33.4-42.8) | 44.2(39.4-50.0) | 0.11(0.110.11) |
| Fiji | 231.8(204.8-262.4) | 41.5(37.0-46.8) | 337.2(298.4-385.0) | 42.0(37.4-47.7) | 0.04(0.030.03) |
| Finland | 2623.2(2338.2-2971.7) | 42.9(38.5-48.2) | 3658.7(3250.9-4150.3) | 43.1(38.5-48.4) | -0.05(-0.08-0.08) |
| France | 27208.8(24190.0-30782.3) | 38.3(33.9-43.1) | 35395.2(31478.2-40197.2) | 36.3(32.4-40.7) | -0.26(-0.30-0.30) |
| Gabon | 294.8(261.1-333.2) | 40.3(35.9-45.7) | 538.5(477.1-607.1) | 40.0(35.6-45.2) | -0.05(-0.07-0.07) |
| Georgia | 1674.4(1485.7-1897.6) | 28.7(25.7-32.4) | 1395.7(1240.0-1580.6) | 29.0(25.9-32.4) | 0.08(0.040.04) |
| Germany | 44389.0(39397.4-50540.3) | 42.2(37.7-47.6) | 51807.3(45842.0-59080.4) | 39.2(35.1-44.0) | -0.35(-0.38-0.38) |
| Ghana | 3863.1(3383.8-4387.0) | 38.8(34.3-43.8) | 8635.9(7635.3-9794.0) | 38.9(34.6-44.1) | 0.01(0.000.00) |
| Greece | 4589.3(4080.0-5207.6) | 35.5(31.7-39.9) | 5868.0(5218.7-6717.0) | 35.4(31.7-40.0) | -0.06(-0.08-0.08) |
| Greenland | 7.5(6.6-8.6) | 16.2(14.4-18.2) | 9.8(8.6-11.2) | 15.9(14.1-17.9) | -0.07(-0.10-0.10) |
| Grenada | 33.6(30.0-37.8) | 43.7(38.9-49.1) | 60.1(53.7-68.2) | 44.1(39.3-50.0) | 0.02(0.010.01) |
| Guam | 49.2(43.6-55.7) | 45.6(40.4-51.6) | 78.4(69.5-88.7) | 45.7(40.9-51.5) | 0.00(-0.02-0.02) |
| Guatemala | 2121.1(1874.2-2380.9) | 39.1(34.8-44.1) | 5493.9(4890.4-6224.0) | 39.9(35.7-45.0) | 0.09(0.070.07) |
| Guinea | 1611.5(1429.8-1816.1) | 36.6(32.8-41.3) | 3031.3(2672.5-3428.2) | 37.6(33.6-42.7) | 0.08(0.070.07) |
| Guinea-Bissau | 238.4(209.6-271.5) | 37.1(33.0-42.1) | 460.5(403.0-523.9) | 38.3(34.1-43.4) | 0.09(0.070.07) |
| Guyana | 246.7(217.2-278.3) | 43.4(38.8-48.8) | 284.8(253.0-321.5) | 42.6(38.1-48.0) | -0.10(-0.12-0.12) |
| Haiti | 1878.4(1657.1-2129.4) | 41.7(36.9-47.1) | 3864.5(3413.1-4359.7) | 42.7(38.1-48.1) | 0.08(0.070.07) |
| Honduras | 1317.1(1171.1-1492.4) | 41.5(36.9-46.9) | 3198.4(2837.2-3611.4) | 42.1(37.4-47.3) | 0.04(0.030.03) |
| Hungary | 7670.9(6844.1-8658.7) | 61.1(54.7-68.3) | 8724.5(7780.3-9891.9) | 62.3(55.8-69.3) | -0.06(-0.25-0.25) |
| Iceland | 93.3(83.2-105.2) | 34.1(30.4-38.5) | 143.5(127.7-162.5) | 33.3(29.6-37.5) | -0.18(-0.22-0.22) |
| India | 241174.4(213626.7-271756.9) | 37.3(33.3-41.9) | 444784.0(395471.2-502974.0) | 36.3(32.4-41.0) | -0.12(-0.15-0.15) |
| Indonesia | 67874.1(60226.7-76616.1) | 48.2(43.2-54.3) | 118581.9(105403.1-133494.0) | 50.8(45.5-57.0) | 0.20(0.200.20) |
| Iran | 14748.9(13005.0-16744.0) | 37.2(33.2-42.1) | 29153.2(25751.9-33119.9) | 37.6(33.5-42.5) | 0.03(0.000.00) |
| Iraq | 4312.9(3803.1-4881.1) | 36.6(32.5-41.4) | 12233.5(10785.7-13936.1) | 37.9(33.8-42.7) | 0.11(0.080.08) |
| Ireland | 1491.0(1329.2-1670.3) | 38.7(34.6-43.4) | 2242.2(1998.8-2534.7) | 37.4(33.4-42.0) | -0.23(-0.28-0.28) |
| Israel | 1679.5(1501.5-1887.3) | 34.2(30.4-38.4) | 3368.8(3008.0-3817.2) | 33.8(30.2-38.2) | -0.11(-0.14-0.14) |
| Italy | 28229.8(25077.9-32044.0) | 38.6(34.4-43.3) | 35261.1(31401.0-40114.6) | 36.1(32.4-40.4) | -0.28(-0.33-0.33) |
| Jamaica | 863.9(770.2-972.5) | 41.8(37.1-47.0) | 1275.4(1139.2-1438.7) | 43.9(39.3-49.5) | 0.20(0.180.18) |
| Japan | 69240.1(61927.0-77959.9) | 47.1(42.2-52.7) | 93900.4(83335.9-106402.1) | 45.4(40.7-50.7) | -0.22(-0.25-0.25) |
| Jordan | 881.1(770.9-1004.2) | 36.2(32.2-41.0) | 3020.0(2657.9-3406.0) | 36.7(32.7-41.2) | 0.05(0.040.04) |
| Kazakhstan | 5894.0(5229.1-6620.9) | 40.0(35.7-45.0) | 6673.8(5941.4-7573.0) | 38.9(34.9-44.0) | -0.05(-0.11-0.11) |
| Kenya | 5698.0(5019.7-6468.0) | 39.3(35.2-44.2) | 13463.5(11904.7-15223.1) | 40.3(35.9-45.4) | 0.06(0.050.05) |
| Kiribati | 22.0(19.4-24.9) | 40.2(36.0-45.5) | 36.8(32.6-41.7) | 40.6(36.3-45.8) | 0.03(0.020.02) |
| Kuwait | 460.9(397.6-531.7) | 36.9(33.0-41.7) | 1341.2(1164.7-1539.5) | 36.9(32.9-42.1) | 0.04(0.010.01) |
| Kyrgyzstan | 1912.0(1703.2-2142.5) | 51.9(46.3-58.3) | 3062.5(2722.8-3427.6) | 57.1(50.7-64.0) | 0.52(0.390.39) |
| Laos | 1369.9(1216.0-1546.7) | 45.3(40.5-51.1) | 2547.1(2249.8-2873.3) | 45.8(40.9-51.7) | 0.03(0.020.02) |
| Latvia | 1103.1(982.5-1245.6) | 34.7(31.2-39.0) | 1285.8(1146.3-1452.1) | 44.2(39.7-49.7) | 1.17(0.890.89) |
| Lebanon | 1162.6(1033.0-1319.8) | 38.4(34.3-43.4) | 2821.5(2508.6-3180.0) | 38.8(34.6-43.6) | 0.07(0.040.04) |
| Lesotho | 514.1(452.1-583.6) | 38.9(34.5-44.1) | 612.7(542.5-694.6) | 39.7(35.3-44.9) | 0.07(0.060.06) |
| Liberia | 555.0(492.6-627.1) | 38.5(34.2-43.5) | 1254.5(1103.0-1431.7) | 39.3(35.0-44.3) | 0.09(0.080.08) |
| Libya | 981.5(866.2-1113.2) | 33.8(30.1-38.2) | 2120.6(1866.3-2401.2) | 36.5(32.4-41.2) | 0.29(0.270.27) |
| Lithuania | 1476.0(1318.0-1654.4) | 35.2(31.4-39.3) | 1623.1(1443.3-1845.3) | 38.3(34.3-42.9) | 0.65(0.410.41) |
| Luxembourg | 182.9(162.6-205.9) | 38.5(34.2-43.1) | 286.9(256.9-325.3) | 37.2(33.3-41.9) | -0.16(-0.19-0.19) |
| Macedonia | 914.6(810.7-1036.6) | 47.3(42.2-53.3) | 1155.2(1026.4-1307.5) | 42.3(37.8-47.7) | -0.48(-0.51-0.51) |
| Madagascar | 2984.7(2636.3-3370.5) | 37.8(33.6-42.9) | 6568.8(5766.6-7467.0) | 38.4(34.2-43.4) | 0.05(0.040.04) |
| Malawi | 2400.1(2118.3-2717.1) | 37.7(33.7-42.4) | 4701.9(4138.9-5299.4) | 40.1(35.5-45.3) | 0.17(0.140.14) |
| Malaysia | 6196.7(5483.5-6987.1) | 46.3(41.2-52.1) | 13655.6(12106.8-15400.9) | 48.7(43.4-54.8) | 0.26(0.210.21) |
| Maldives | 63.3(55.8-72.3) | 42.4(37.8-48.1) | 173.5(153.2-198.0) | 44.8(40.1-50.6) | 0.16(0.120.12) |
| Mali | 2085.2(1835.5-2357.0) | 36.2(32.2-40.8) | 4769.4(4206.9-5386.9) | 36.4(32.5-40.9) | 0.00(-0.02-0.02) |
| Malta | 148.1(131.8-167.9) | 37.0(32.9-41.7) | 220.8(195.5-252.6) | 34.2(30.6-38.5) | -0.36(-0.43-0.43) |
| Marshall Islands | 13.0(11.4-14.8) | 42.6(38.1-48.3) | 18.9(16.7-21.4) | 43.6(38.8-49.1) | 0.08(0.070.07) |
| Mauritania | 554.5(490.5-629.8) | 38.3(34.0-43.5) | 1065.2(939.9-1208.0) | 37.8(33.7-43.0) | -0.09(-0.11-0.11) |
| Mauritius | 438.0(388.1-494.4) | 47.4(42.3-53.5) | 686.8(606.7-783.1) | 47.1(42.0-53.2) | -0.03(-0.04-0.04) |
| Mexico | 26976.6(23951.9-30325.9) | 42.9(38.4-48.4) | 51419.6(45809.1-58024.9) | 42.6(38.1-48.0) | -0.05(-0.07-0.07) |
| Moldova | 1318.8(1166.7-1489.0) | 29.9(26.6-33.8) | 1730.7(1544.1-1964.7) | 37.1(33.2-41.7) | 1.05(0.870.87) |
| Mongolia | 500.4(440.8-565.4) | 32.1(28.6-36.2) | 840.3(743.8-952.7) | 31.3(27.8-35.3) | -0.16(-0.19-0.19) |
| Montenegro | 396.5(353.9-444.6) | 63.1(56.3-70.6) | 491.3(437.5-554.1) | 62.0(55.4-69.2) | -0.02(-0.06-0.06) |
| Morocco | 7256.5(6407.1-8222.7) | 37.7(33.5-42.7) | 13446.0(11880.4-15111.1) | 40.0(35.4-45.0) | 0.20(0.200.20) |
| Mozambique | 3697.2(3244.9-4181.9) | 38.9(34.6-44.1) | 7704.9(6748.0-8802.3) | 40.2(35.9-45.5) | 0.11(0.100.10) |
| Myanmar | 14718.5(13069.9-16513.9) | 46.9(41.8-52.5) | 23008.8(20386.0-26084.9) | 47.4(42.3-53.6) | 0.04(0.020.02) |
| Namibia | 393.1(349.2-443.5) | 38.8(34.6-43.9) | 728.5(643.8-825.2) | 39.2(34.9-44.5) | -0.02(-0.05-0.05) |
| Nepal | 4542.5(4011.7-5157.3) | 33.5(29.9-37.8) | 8608.7(7673.4-9748.6) | 33.9(30.3-38.3) | 0.05(0.020.02) |
| Netherlands | 6736.3(5983.3-7602.7) | 37.9(33.9-42.8) | 9145.9(8112.7-10393.8) | 37.2(33.1-41.9) | -0.16(-0.20-0.20) |
| New Zealand | 1692.3(1505.1-1893.3) | 45.3(40.4-50.5) | 2652.1(2376.0-2993.8) | 44.5(40.0-49.6) | -0.10(-0.13-0.13) |
| Nicaragua | 1064.2(937.5-1205.5) | 41.4(37.0-47.0) | 2439.5(2172.4-2742.5) | 44.6(39.9-50.4) | 0.30(0.280.28) |
| Niger | 1780.9(1560.4-2029.6) | 36.4(32.4-40.9) | 4731.0(4171.5-5373.4) | 36.8(32.8-41.6) | 0.00(-0.01-0.01) |
| Nigeria | 23741.7(21044.2-26807.3) | 37.3(33.4-42.2) | 52608.6(46468.1-59736.0) | 38.6(34.4-43.5) | 0.12(0.090.09) |
| North Korea | 7211.7(6431.2-8125.6) | 39.9(35.6-44.9) | 11110.1(9860.9-12568.3) | 39.7(35.5-44.7) | -0.03(-0.05-0.05) |
| Northern Mariana Islands | 15.0(13.0-17.2) | 46.1(41.2-51.9) | 22.4(19.6-25.5) | 47.1(42.1-53.0) | 0.10(0.080.08) |
| Norway | 2537.1(2256.5-2874.1) | 46.8(41.8-52.6) | 3209.3(2858.3-3621.8) | 45.2(40.6-50.6) | -0.20(-0.24-0.24) |
| Oman | 434.8(380.2-494.8) | 34.5(30.8-39.0) | 1384.7(1185.6-1614.7) | 39.1(34.8-44.2) | 0.58(0.530.53) |
| Pakistan | 27013.6(23964.4-30553.7) | 34.6(30.8-39.2) | 54713.5(48210.2-62142.8) | 34.6(30.8-39.1) | 0.00(-0.02-0.02) |
| Palestine | 485.2(429.0-549.9) | 35.7(31.9-40.4) | 1312.6(1158.9-1481.5) | 37.0(33.0-42.0) | 0.10(0.080.08) |
| Panama | 822.4(729.1-921.7) | 42.7(38.1-48.1) | 1712.1(1526.9-1933.1) | 43.2(38.6-48.9) | 0.06(0.050.05) |
| Papua New Guinea | 1195.0(1058.0-1361.6) | 42.4(37.9-48.0) | 2853.9(2513.1-3240.0) | 43.4(38.8-49.1) | 0.08(0.050.05) |
| Paraguay | 1295.0(1149.6-1465.1) | 43.0(38.4-48.7) | 2706.8(2406.5-3066.1) | 44.4(39.6-50.1) | 0.12(0.110.11) |
| Peru | 6233.4(5528.7-7006.2) | 37.9(33.9-42.9) | 12823.2(11465.9-14435.1) | 40.1(35.8-45.2) | 0.20(0.170.17) |
| Philippines | 20966.0(18566.1-23769.8) | 45.4(40.6-51.1) | 39148.5(34776.8-44105.6) | 45.2(40.3-50.6) | -0.06(-0.08-0.08) |
| Poland | 18720.7(16751.4-21199.6) | 44.9(40.2-50.9) | 24709.2(21912.6-27990.8) | 47.2(42.1-53.0) | 0.27(0.220.22) |
| Portugal | 4289.8(3828.2-4862.5) | 35.5(31.8-40.0) | 5869.9(5198.1-6716.0) | 34.7(31.0-39.2) | -0.16(-0.22-0.22) |
| Puerto Rico | 1592.2(1420.7-1798.8) | 43.2(38.6-48.9) | 2379.7(2119.3-2718.0) | 44.4(39.7-49.9) | 0.10(0.080.08) |
| Qatar | 121.6(103.4-141.8) | 39.6(35.1-45.0) | 847.3(711.0-991.5) | 39.0(34.8-44.0) | -0.12(-0.15-0.15) |
| Romania | 10266.1(9125.1-11593.9) | 40.3(36.0-45.3) | 10458.9(9244.3-11849.8) | 37.8(33.7-42.5) | -0.29(-0.31-0.31) |
| Russian Federation | 110710.4(98895.1-124273.1) | 67.0(60.2-75.1) | 103526.6(91773.7-116883.4) | 55.1(49.1-61.8) | -0.86(-1.04-1.04) |
| Rwanda | 1801.3(1587.3-2046.2) | 38.5(34.4-43.6) | 3306.9(2918.4-3765.5) | 37.9(33.7-43.0) | -0.12(-0.18-0.18) |
| Saint Lucia | 49.9(44.3-56.4) | 44.9(40.0-51.0) | 89.8(80.1-101.8) | 45.8(40.9-51.8) | 0.08(0.040.04) |
| Saint Vincent and the Grenadines | 40.4(36.0-45.5) | 44.7(40.0-50.6) | 56.6(50.5-64.5) | 44.5(39.7-50.6) | 0.00(-0.02-0.02) |
| Samoa | 52.9(46.7-59.9) | 43.3(38.6-48.7) | 73.0(65.1-82.7) | 44.5(39.7-50.4) | 0.10(0.090.09) |
| Sao Tome and Principe | 33.8(30.1-38.0) | 38.4(34.0-43.2) | 59.7(52.8-67.4) | 40.5(36.1-45.7) | 0.18(0.150.15) |
| Saudi Arabia | 4136.6(3650.3-4677.4) | 38.1(33.7-43.2) | 11126.4(9633.8-12768.0) | 40.7(36.1-45.9) | 0.24(0.220.22) |
| Senegal | 1926.1(1701.6-2176.8) | 38.4(34.2-43.4) | 4072.8(3600.9-4577.2) | 39.0(34.8-44.1) | 0.04(0.030.03) |
| Serbia | 4871.3(4324.3-5532.8) | 47.7(42.3-53.7) | 5144.0(4590.6-5837.8) | 42.5(38.3-47.6) | -0.47(-0.49-0.49) |
| Seychelles | 38.3(33.9-42.7) | 58.4(52.3-65.5) | 56.6(50.5-63.8) | 54.7(49.0-61.5) | -0.37(-0.42-0.42) |
| Sierra Leone | 1029.8(914.6-1154.9) | 38.2(34.1-43.2) | 2114.3(1862.1-2401.4) | 39.3(35.1-44.3) | 0.11(0.080.08) |
| Singapore | 1053.7(925.0-1196.1) | 36.3(32.3-41.0) | 2280.7(2022.7-2600.3) | 36.3(32.4-41.0) | -0.10(-0.14-0.14) |
| Slovakia | 2627.4(2339.4-2953.7) | 46.7(41.7-52.6) | 3394.6(3004.0-3842.2) | 48.3(43.2-54.2) | 0.15(0.100.10) |
| Slovenia | 997.6(889.1-1128.1) | 44.9(40.3-50.7) | 1179.3(1048.9-1345.7) | 38.7(34.7-43.7) | -0.63(-0.67-0.67) |
| Solomon Islands | 99.8(87.5-113.1) | 43.5(38.7-48.9) | 203.6(180.3-229.6) | 43.6(39.0-49.3) | 0.01(0.010.01) |
| Somalia | 1640.8(1443.1-1879.5) | 37.0(33.0-42.0) | 4047.9(3533.3-4601.7) | 37.9(33.6-42.8) | 0.07(0.060.06) |
| South Africa | 11467.2(10239.8-12873.8) | 40.4(36.1-45.5) | 20395.1(18081.4-23075.3) | 41.2(36.8-46.4) | 0.03(0.000.00) |
| South Korea | 13662.1(12031.1-15461.6) | 35.1(31.3-39.7) | 23539.6(20818.7-26865.3) | 35.0(31.2-39.7) | -0.06(-0.09-0.09) |
| South Sudan | 1390.3(1221.1-1586.9) | 36.1(32.2-40.7) | 2338.8(2061.5-2656.5) | 37.2(33.2-42.0) | 0.07(0.050.05) |
| Spain | 17316.1(15445.9-19728.0) | 36.1(32.3-40.8) | 24908.5(22093.0-28228.9) | 35.7(31.9-40.1) | -0.13(-0.16-0.16) |
| Sri Lanka | 6296.7(5584.6-7121.6) | 44.9(40.1-50.8) | 10645.9(9395.9-12094.4) | 46.5(41.2-52.6) | 0.06(0.040.04) |
| Sudan | 5230.2(4627.0-5918.1) | 37.8(33.8-42.8) | 10769.3(9518.4-12201.6) | 38.5(34.2-43.6) | 0.04(0.010.01) |
| Suriname | 129.6(114.9-146.4) | 40.8(36.4-46.0) | 238.3(210.8-271.4) | 41.4(36.8-46.8) | 0.06(0.050.05) |
| Swaziland | 202.8(178.0-230.9) | 39.7(35.4-44.8) | 325.1(285.4-369.3) | 39.5(35.3-44.8) | -0.04(-0.06-0.06) |
| Sweden | 5412.9(4806.5-6104.8) | 46.1(41.3-51.8) | 6719.6(5977.4-7601.7) | 45.2(40.6-50.7) | -0.11(-0.12-0.12) |
| Switzerland | 2849.0(2525.1-3215.2) | 32.2(28.7-36.1) | 3796.0(3374.1-4314.0) | 30.2(27.0-33.8) | -0.29(-0.32-0.32) |
| Syria | 3121.9(2747.7-3530.6) | 37.1(33.0-42.2) | 5906.5(5266.9-6666.5) | 38.8(34.5-43.8) | 0.13(0.110.11) |
| Taiwan (Province of China) | 7827.1(6955.3-8829.3) | 43.1(38.5-48.7) | 12645.4(11213.5-14349.3) | 41.6(37.1-46.8) | -0.15(-0.16-0.16) |
| Tajikistan | 1325.1(1170.8-1490.8) | 33.4(29.7-37.6) | 2295.4(2025.2-2605.8) | 32.4(28.9-36.6) | -0.13(-0.14-0.14) |
| Tanzania | 6346.6(5581.8-7208.9) | 37.3(33.1-42.3) | 14433.5(12744.3-16324.7) | 39.2(35.0-44.4) | 0.19(0.180.18) |
| Thailand | 18694.6(16599.1-21096.1) | 39.9(35.8-45.0) | 35051.4(31134.4-40082.0) | 41.5(36.9-47.0) | 0.17(0.150.15) |
| The Bahamas | 95.5(84.4-108.1) | 46.0(41.0-51.9) | 172.3(153.3-194.9) | 45.6(40.7-51.6) | -0.03(-0.04-0.04) |
| The Gambia | 239.9(210.8-272.0) | 38.7(34.5-43.8) | 575.4(505.1-654.4) | 39.3(34.9-44.2) | 0.05(0.040.04) |
| Timor-Leste | 235.9(207.8-267.1) | 45.2(40.5-50.6) | 486.4(431.4-548.9) | 47.2(42.1-53.3) | 0.15(0.140.14) |
| Togo | 858.5(744.0-972.1) | 37.9(33.6-42.8) | 2016.4(1770.9-2285.1) | 38.4(34.1-43.5) | 0.03(0.020.02) |
| Tonga | 32.2(28.6-36.4) | 43.2(38.6-48.7) | 40.1(35.7-45.1) | 44.2(39.5-49.8) | 0.09(0.090.09) |
| Trinidad and Tobago | 436.0(388.6-491.4) | 42.6(38.1-48.1) | 692.9(614.1-789.2) | 43.2(38.4-48.9) | 0.06(0.050.05) |
| Tunisia | 2379.7(2098.1-2685.0) | 36.2(32.1-40.8) | 4546.3(4008.8-5155.7) | 38.2(33.9-43.1) | 0.19(0.170.17) |
| Turkey | 16641.7(14744.8-18787.3) | 36.4(32.4-41.3) | 30546.7(27223.4-34636.0) | 35.8(31.9-40.5) | -0.13(-0.15-0.15) |
| Turkmenistan | 908.2(801.5-1024.2) | 33.3(29.6-37.5) | 1379.7(1222.4-1560.6) | 31.9(28.5-35.9) | -0.29(-0.35-0.35) |
| Uganda | 4359.5(3830.3-4952.1) | 40.0(35.6-45.2) | 9536.6(8379.2-10859.6) | 39.1(34.9-44.2) | -0.13(-0.15-0.15) |
| Ukraine | 24168.4(21520.8-27473.9) | 39.0(34.8-44.2) | 23481.6(20853.6-26674.1) | 38.5(34.4-43.4) | 0.00(-0.03-0.03) |
| United Arab Emirates | 534.4(457.5-623.7) | 42.7(38.0-48.1) | 3239.0(2674.8-3825.2) | 40.5(36.1-45.6) | -0.20(-0.23-0.23) |
| United Kingdom | 28884.4(25718.2-32633.0) | 38.7(34.7-43.5) | 34156.7(30458.2-38638.5) | 36.3(32.5-40.8) | -0.37(-0.43-0.43) |
| United States | 52466.5(46326.3-59779.7) | 17.8(15.7-20.3) | 69179.6(63734.9-75482.7) | 16.1(14.8-17.5) | -0.56(-0.67-0.67) |
| Uruguay | 850.1(758.7-962.8) | 23.9(21.4-27.1) | 1053.6(939.2-1192.5) | 23.7(21.1-26.7) | -0.15(-0.22-0.22) |
| Uzbekistan | 4244.3(3740.4-4777.8) | 27.0(24.0-30.4) | 7346.8(6487.9-8314.4) | 27.5(24.6-30.8) | 0.06(0.050.05) |
| Vanuatu | 48.2(42.7-54.6) | 45.7(40.7-51.5) | 102.0(90.6-115.2) | 45.9(41.1-51.8) | 0.02(0.010.01) |
| Venezuela | 6485.4(5729.3-7304.8) | 46.2(41.2-51.9) | 13603.5(11995.1-15399.0) | 46.1(40.8-52.1) | 0.00(-0.03-0.03) |
| Vietnam | 24392.8(21637.5-27529.5) | 45.1(40.2-51.0) | 44991.7(39738.1-51030.4) | 48.1(42.7-54.4) | 0.29(0.270.27) |
| Virgin Islands, U.S. | 45.0(39.9-50.6) | 47.2(42.1-53.1) | 68.5(60.4-78.2) | 47.8(42.7-53.5) | 0.06(0.050.05) |
| Yemen | 3091.7(2709.7-3533.4) | 36.7(32.6-41.6) | 7924.0(6966.9-8962.1) | 38.4(34.3-43.5) | 0.17(0.160.16) |
| Zambia | 1786.3(1564.9-2030.9) | 36.1(32.2-41.0) | 4311.8(3758.7-4912.1) | 38.5(34.2-43.4) | 0.19(0.140.14) |
| Zimbabwe | 2562.5(2247.9-2885.0) | 37.9(33.7-42.8) | 3760.9(3304.3-4278.9) | 36.7(32.8-41.6) | -0.18(-0.23-0.23) |
