# Supplementary material for: Global, Regional, and National Burden of Myocarditis From 1990 to 2017: A Systematic Analysis Based on the Global Burden of Disease Study 2017
Source: Front Cardiovasc Med. 2021 Jul 2;8:692990. doi: 10.3389/fcvm.2021.692990 (PMC8284556; doi:10.3389/fcvm.2021.692990)
Supplement: Supplementary file 3 [file Table_3.docx]

**Table S3** The death cases and age-standardized death rate of myocarditis between 1990 and 2017, and its temporal trends from 1990 to 2017 in 195 countries and territories.

|  | 1990 |  | 2017 |  | 1990–2017 |
| --- | --- | --- | --- | --- | --- |
| Countries and territories | Death cases No. (95% UI) | ASDR per 100,000 No. (95% UI) | Death cases No. (95% UI) | ASDR per 100,000 No. (95% UI) | EAPC No. (95% CI) |
| Afghanistan | 30.2(12.4-54.5) | 0.4(0.2-0.7) | 68.1(45.6-107.3) | 0.4(0.2-0.5) | 0.12(-0.24-0.48) |
| Albania | 181.0(134.0-210.2) | 8.8(6.4-10.4) | 162.3(126.0-205.3) | 4.3(3.4-5.4) | -4.03(-6.20--1.81) |
| Algeria | 86.8(60.8-117.1) | 0.4(0.3-0.6) | 117.8(75.4-179.5) | 0.3(0.2-0.5) | -0.17(-2.51-2.22) |
| American Samoa | 0.2(0.1-0.0) | 0.7(0.6-1.0) | 0.3(0.2-0.3) | 0.7(0.5-0.8) | 0.09(-0.66-0.85) |
| Andorra | 0.3(0.2-0.0) | 0.6(0.4-0.8) | 0.9(0.6-1.2) | 0.5(0.4-0.7) | -0.09(-0.32-0.13) |
| Angola | 41.5(21.6-65.6) | 0.6(0.3-0.9) | 56.8(34.3-100.4) | 0.4(0.2-0.7) | -1.56(-1.83--1.28) |
| Antigua and Barbuda | 0.9(0.7-1.1) | 1.7(1.3-2.0) | 1.6(1.3-2.0) | 1.6(1.3-2.1) | -0.63(-1.93-0.68) |
| Argentina | 190.4(138.7-242.2) | 0.6(0.5-0.8) | 201.3(166.1-252.8) | 0.4(0.3-0.5) | -1.16(-2.36-0.06) |
| Armenia | 4.2(1.8-6.6) | 0.2(0.1-0.3) | 5.6(4.1-11.3) | 0.2(0.1-0.3) | -0.52(-1.60-0.58) |
| Australia | 67.5(56.0-103.1) | 0.4(0.3-0.6) | 98.6(82.5-124.2) | 0.3(0.2-0.4) | -1.65(-2.64--0.65) |
| Austria | 179.6(62.8-640.6) | 1.6(0.6-5.6) | 133.6(94.2-263.5) | 0.6(0.5-1.2) | -3.75(-4.53--2.96) |
| Azerbaijan | 84.9(65.6-121.1) | 1.5(1.1-2.1) | 122.3(92.3-181.0) | 1.4(1.1-1.9) | -1.29(-2.23--0.33) |
| Bahrain | 0.7(0.5-0.0) | 0.2(0.1-0.3) | 1.0(0.7-1.2) | 0.1(0.1-0.1) | -3.23(-5.47--0.94) |
| Bangladesh | 445.1(249.7-720.7) | 0.6(0.3-1.0) | 532.6(305.1-844.9) | 0.4(0.3-0.7) | -1.10(-2.20-0.02) |
| Barbados | 3.0(2.3-3.3) | 1.0(0.8-1.2) | 4.2(3.3-5.3) | 0.9(0.7-1.1) | -0.41(-1.01-0.20) |
| Belarus | 38.6(23.1-55.5) | 0.3(0.2-0.5) | 30.8(16.3-57.0) | 0.2(0.1-0.4) | -1.47(-2.74--0.17) |
| Belgium | 60.4(38.8-138.1) | 0.4(0.3-1.0) | 165.2(98.0-215.7) | 0.6(0.4-0.7) | 0.87(-0.46-2.22) |
| Belize | 1.3(1.0-1.1) | 1.2(0.9-1.5) | 3.2(2.6-4.2) | 1.2(1.0-1.6) | -0.58(-1.45-0.30) |
| Benin | 21.4(12.3-32.3) | 0.8(0.4-1.2) | 24.5(16.7-35.3) | 0.4(0.3-0.6) | -1.89(-2.84--0.93) |
| Bermuda | 0.3(0.2-0.0) | 0.6(0.4-0.8) | 0.5(0.4-0.7) | 0.4(0.4-0.6) | -1.07(-1.35--0.78) |
| Bhutan | 2.3(1.4-3.3) | 0.7(0.4-1.1) | 3.2(1.9-5.6) | 0.5(0.3-0.9) | -1.32(-1.45--1.19) |
| Bolivia | 29.4(17.9-42.4) | 0.7(0.4-1.0) | 31.4(22.8-42.8) | 0.4(0.3-0.5) | -2.43(-2.63--2.23) |
| Bosnia and Herzegovina | 49.2(27.3-72.7) | 1.5(0.9-2.3) | 119.3(67.2-169.1) | 2.4(1.4-3.4) | 1.56(0.15-2.98) |
| Botswana | 3.8(2.7-5.5) | 0.5(0.3-0.6) | 5.1(3.4-7.2) | 0.3(0.2-0.5) | -0.41(-2.20-1.42) |
| Brazil | 356.4(300.5-536.5) | 0.3(0.3-0.5) | 647.8(541.1-968.8) | 0.3(0.3-0.5) | -0.48(-0.86--0.09) |
| Brunei | 2.1(1.4-2.2) | 1.2(0.8-1.4) | 3.3(2.8-3.9) | 0.9(0.8-1.1) | -1.57(-2.66--0.48) |
| Bulgaria | 121.0(87.8-191.1) | 1.2(0.9-1.9) | 270.6(230.6-330.5) | 2.0(1.7-2.4) | 0.75(-0.35-1.85) |
| Burkina Faso | 38.6(22.9-60.6) | 0.7(0.5-1.2) | 47.9(32.4-68.9) | 0.5(0.3-0.7) | -1.38(-2.71--0.03) |
| Burundi | 23.2(12.4-41.4) | 0.6(0.3-1.1) | 18.6(8.5-36.5) | 0.3(0.1-0.6) | -2.94(-3.48--2.40) |
| Cambodia | 43.8(24.0-71.7) | 0.6(0.3-0.9) | 65.0(46.1-87.9) | 0.6(0.4-0.8) | -0.27(-0.98-0.43) |
| Cameroon | 53.4(35.9-72.7) | 1.0(0.7-1.4) | 69.5(52.1-93.7) | 0.5(0.4-0.7) | -2.90(-3.17--2.64) |
| Canada | 31.4(23.2-51.5) | 0.1(0.1-0.2) | 88.4(48.4-115.5) | 0.2(0.1-0.2) | 2.96(1.71-4.22) |
| Cape Verde | 0.7(0.5-0.0) | 0.2(0.2-0.3) | 1.3(1.1-1.7) | 0.3(0.2-0.3) | -0.08(-0.32-0.17) |
| Central African Republic | 10.8(5.5-17.1) | 0.6(0.3-1.0) | 13.9(7.4-25.3) | 0.5(0.2-0.8) | -1.34(-2.01--0.66) |
| Chad | 26.2(14.0-44.4) | 0.7(0.4-1.3) | 33.0(21.8-49.7) | 0.4(0.3-0.6) | -2.31(-2.50--2.12) |
| Chile | 14.2(12.2-18.1) | 0.1(0.1-0.2) | 28.7(23.9-35.6) | 0.1(0.1-0.2) | 0.91(-0.19-2.01) |
| China | 7198.0(5776.0-8211.8) | 0.8(0.7-0.9) | 14766.9(12242.2-16134.2) | 1.0(0.9-1.1) | 0.77(-1.18-2.75) |
| Colombia | 73.8(54.1-85.8) | 0.3(0.2-0.3) | 101.1(79.0-140.7) | 0.2(0.2-0.3) | -0.55(-2.10-1.02) |
| Comoros | 1.8(1.1-2.2) | 0.5(0.3-0.9) | 1.4(0.5-2.7) | 0.3(0.1-0.6) | -2.83(-3.13--2.53) |
| Congo | 8.9(5.9-14.1) | 0.6(0.4-1.0) | 13.7(6.9-25.3) | 0.5(0.2-0.9) | -1.37(-1.59--1.14) |
| Costa Rica | 8.7(6.1-10.1) | 0.4(0.3-0.4) | 15.5(12.4-22.7) | 0.3(0.2-0.4) | -0.94(-1.36--0.53) |
| Cote d'Ivoire | 44.7(28.2-61.6) | 0.7(0.4-1.0) | 63.2(44.6-90.1) | 0.5(0.3-0.7) | -1.90(-2.11--1.69) |
| Croatia | 132.3(104.0-154.1) | 2.6(2.0-3.0) | 278.0(192.0-344.5) | 3.2(2.3-3.9) | -0.77(-3.72-2.28) |
| Cuba | 54.3(44.6-74.7) | 0.5(0.5-0.8) | 97.0(70.9-170.0) | 0.5(0.4-0.9) | -0.18(-0.65-0.28) |
| Cyprus | 9.6(5.6-13.1) | 1.4(0.8-1.9) | 14.7(8.9-19.3) | 0.8(0.5-1.0) | -2.19(-2.69--1.69) |
| Czech Republic | 60.1(34.3-132.1) | 0.5(0.3-1.1) | 189.1(138.4-223.3) | 1.0(0.7-1.1) | 1.92(1.42-2.42) |
| Democratic Republic of the Congo | 118.1(69.9-178.1) | 0.5(0.3-0.7) | 164.5(86.9-269.5) | 0.3(0.2-0.6) | -1.24(-1.40--1.07) |
| Denmark | 67.8(44.5-84.8) | 0.9(0.6-1.0) | 70.1(53.8-82.9) | 0.6(0.5-0.7) | -1.80(-2.10--1.49) |
| Djibouti | 1.8(0.9-3.3) | 0.6(0.3-1.1) | 2.3(0.9-5.1) | 0.3(0.1-0.8) | -2.60(-2.83--2.37) |
| Dominica | 1.0(0.6-1.1) | 1.3(0.9-1.7) | 1.2(0.9-1.5) | 1.2(1.0-1.6) | -0.47(-0.55--0.39) |
| Dominican Republic | 43.6(32.0-54.5) | 1.1(0.8-1.5) | 73.3(57.4-90.6) | 0.8(0.6-1.0) | -1.30(-1.65--0.95) |
| Ecuador | 23.9(14.2-30.3) | 0.4(0.2-0.5) | 34.4(26.8-49.3) | 0.2(0.2-0.3) | -2.26(-2.50--2.02) |
| Egypt | 279.9(176.2-389.3) | 0.5(0.4-0.7) | 230.3(159.3-343.2) | 0.3(0.2-0.5) | -2.03(-2.14--1.93) |
| El Salvador | 14.3(11.6-17.1) | 0.4(0.3-0.5) | 23.1(15.4-30.5) | 0.4(0.3-0.5) | 0.30(0.09-0.51) |
| Equatorial Guinea | 1.8(0.9-2.2) | 0.6(0.3-0.9) | 2.2(1.0-4.7) | 0.4(0.2-0.8) | -2.01(-2.20--1.81) |
| Eritrea | 14.8(7.1-26.2) | 0.8(0.4-1.5) | 17.3(5.1-33.7) | 0.5(0.1-1.0) | -2.48(-2.74--2.23) |
| Estonia | 2.3(1.8-2.2) | 0.1(0.1-0.2) | 3.4(2.5-5.1) | 0.1(0.1-0.2) | 0.59(0.19-0.98) |
| Ethiopia | 193.9(113.7-316.3) | 0.5(0.3-0.8) | 149.4(66.3-263.8) | 0.3(0.1-0.5) | -2.73(-2.93--2.54) |
| Federated States of Micronesia | 1.5(1.0-2.2) | 2.8(1.9-4.1) | 1.7(1.1-2.4) | 2.5(1.8-3.6) | -0.48(-0.63--0.33) |
| Fiji | 18.8(15.5-22.2) | 5.0(4.0-6.1) | 25.6(20.8-32.4) | 3.8(3.1-4.8) | -1.28(-1.69--0.87) |
| Finland | 72.7(49.7-89.8) | 1.2(0.8-1.5) | 68.6(51.2-106.6) | 0.7(0.5-0.9) | -2.47(-2.75--2.18) |
| France | 102.3(47.9-306.3) | 0.1(0.1-0.4) | 424.7(249.2-573.0) | 0.2(0.1-0.3) | 3.65(2.78-4.53) |
| Gabon | 3.6(2.4-6.6) | 0.6(0.4-1.0) | 4.7(2.6-9.4) | 0.4(0.2-0.9) | -1.27(-1.43--1.11) |
| Georgia | 55.7(39.1-69.6) | 0.9(0.7-1.2) | 64.8(21.4-92.0) | 1.3(0.4-1.8) | 1.15(0.51-1.80) |
| Germany | 1182.6(768.1-1573.1) | 1.0(0.7-1.3) | 1416.6(1080.4-2311.8) | 0.7(0.5-1.0) | -2.00(-2.54--1.46) |
| Ghana | 59.6(30.9-85.8) | 0.8(0.4-1.2) | 90.4(68.9-120.6) | 0.7(0.5-0.9) | -1.15(-1.49--0.80) |
| Greece | 121.0(34.8-177.1) | 0.9(0.3-1.3) | 108.5(70.1-145.1) | 0.4(0.3-0.5) | -2.60(-4.99--0.15) |
| Greenland | 0.4(0.3-0.0) | 0.9(0.7-1.1) | 0.4(0.2-0.5) | 0.6(0.4-0.8) | -1.08(-1.43--0.72) |
| Grenada | 2.4(1.8-3.3) | 3.2(2.4-4.0) | 3.2(2.5-4.1) | 1.9(1.6-2.4) | -1.95(-2.30--1.60) |
| Guam | 1.0(0.8-1.1) | 1.2(1.0-1.6) | 1.8(1.5-2.1) | 1.1(0.9-1.3) | -0.48(-0.83--0.12) |
| Guatemala | 16.8(12.4-20.2) | 0.3(0.2-0.4) | 41.9(34.2-49.9) | 0.3(0.3-0.4) | -0.06(-0.24-0.11) |
| Guinea | 36.0(19.1-56.5) | 0.9(0.4-1.4) | 30.8(20.5-44.1) | 0.5(0.3-0.7) | -2.55(-2.86--2.24) |
| Guinea-Bissau | 5.6(3.5-8.8) | 1.1(0.7-1.6) | 4.8(3.5-6.5) | 0.5(0.4-0.7) | -3.10(-3.40--2.81) |
| Guyana | 8.6(6.5-12.1) | 2.1(1.6-3.0) | 17.1(13.9-20.3) | 3.1(2.5-3.6) | 1.46(1.15-1.77) |
| Haiti | 90.4(55.4-142.1) | 2.4(1.4-3.6) | 117.6(75.8-172.3) | 1.8(1.2-2.7) | -0.91(-0.99--0.84) |
| Honduras | 19.2(13.9-25.2) | 0.7(0.5-0.9) | 29.4(19.8-43.7) | 0.5(0.3-0.7) | -1.24(-1.52--0.95) |
| Hungary | 107.5(85.6-183.1) | 0.9(0.7-1.5) | 202.5(121.8-252.6) | 1.0(0.6-1.3) | 2.34(1.35-3.35) |
| Iceland | 1.2(0.8-2.2) | 0.4(0.3-0.8) | 2.8(2.1-3.4) | 0.4(0.3-0.5) | 0.38(-0.36-1.13) |
| India | 2780.7(1742.3-4492.4) | 0.5(0.3-0.8) | 5401.2(3321.5-8012.3) | 0.5(0.3-0.8) | 0.10(-0.11-0.30) |
| Indonesia | 596.2(422.6-779.7) | 0.4(0.3-0.6) | 982.4(636.2-1595.4) | 0.5(0.3-0.8) | 0.30(0.12-0.47) |
| Iran | 173.7(129.3-210.2) | 0.4(0.3-0.4) | 231.0(197.5-286.5) | 0.3(0.3-0.4) | 0.62(0.08-1.15) |
| Iraq | 279.0(189.8-382.3) | 1.7(1.2-2.2) | 327.9(269.0-399.6) | 0.9(0.7-1.0) | -2.44(-2.79--2.10) |
| Ireland | 15.4(8.4-45.4) | 0.4(0.2-1.3) | 70.5(27.8-102.1) | 0.9(0.4-1.3) | 4.57(3.44-5.71) |
| Israel | 17.0(13.7-23.2) | 0.4(0.3-0.5) | 31.3(23.6-47.1) | 0.3(0.2-0.4) | -1.59(-2.06--1.11) |
| Italy | 216.1(61.2-974.9) | 0.3(0.1-1.2) | 1619.9(380.6-2549.3) | 0.8(0.2-1.2) | 7.99(4.74-11.35) |
| Jamaica | 12.9(9.8-15.1) | 0.7(0.5-0.8) | 17.4(13.2-24.1) | 0.6(0.4-0.8) | -1.61(-2.12--1.09) |
| Japan | 1451.9(1045.0-1604.1) | 1.0(0.7-1.1) | 1530.8(1209.7-2368.1) | 0.4(0.3-0.6) | -3.41(-3.76--3.06) |
| Jordan | 10.0(7.6-12.1) | 0.4(0.3-0.4) | 12.9(10.8-15.3) | 0.2(0.2-0.2) | -3.17(-3.62--2.72) |
| Kazakhstan | 18.8(13.0-40.4) | 0.1(0.1-0.3) | 265.3(191.1-331.6) | 1.7(1.1-2.0) | 13.36(11.12-15.65) |
| Kenya | 50.0(29.9-82.8) | 0.4(0.2-0.7) | 79.5(31.7-157.4) | 0.3(0.1-0.6) | -1.23(-1.41--1.05) |
| Kiribati | 1.8(1.5-2.2) | 4.1(3.3-5.8) | 3.0(2.2-4.4) | 4.0(3.0-5.8) | -0.05(-0.11-0.00) |
| Kuwait | 6.4(5.4-7.7) | 0.4(0.3-0.5) | 9.7(8.0-11.5) | 0.2(0.2-0.3) | -1.51(-1.84--1.18) |
| Kyrgyzstan | 14.7(8.8-19.1) | 0.4(0.2-0.6) | 19.1(11.4-25.4) | 0.3(0.2-0.4) | -1.19(-1.55--0.83) |
| Laos | 39.8(21.6-61.6) | 0.9(0.6-1.4) | 42.0(28.9-60.3) | 0.8(0.5-1.2) | -0.71(-0.94--0.47) |
| Latvia | 3.4(2.5-4.4) | 0.1(0.1-0.1) | 3.4(1.9-7.0) | 0.1(0.1-0.2) | -0.78(-1.21--0.34) |
| Lebanon | 9.0(7.2-11.1) | 0.3(0.2-0.4) | 12.3(8.3-18.3) | 0.2(0.1-0.3) | -2.28(-2.55--2.01) |
| Lesotho | 6.4(3.9-9.9) | 0.5(0.3-0.8) | 7.2(4.8-10.4) | 0.5(0.4-0.7) | 0.44(0.25-0.63) |
| Liberia | 9.3(5.9-13.1) | 0.7(0.4-1.0) | 8.6(5.7-11.8) | 0.3(0.2-0.5) | -3.22(-3.64--2.80) |
| Libya | 39.4(27.3-54.5) | 0.8(0.6-1.0) | 21.6(13.6-33.0) | 0.4(0.2-0.6) | -2.97(-3.17--2.77) |
| Lithuania | 4.4(3.2-7.7) | 0.1(0.1-0.2) | 5.9(4.2-10.4) | 0.1(0.1-0.2) | 0.36(0.05-0.68) |
| Luxembourg | 4.3(2.9-8.8) | 0.9(0.6-1.8) | 12.5(8.8-15.9) | 1.1(0.8-1.4) | 0.90(0.12-1.69) |
| Macedonia | 6.3(4.0-8.8) | 0.4(0.3-0.5) | 10.1(6.4-13.9) | 0.4(0.2-0.5) | 0.06(-0.21-0.33) |
| Madagascar | 74.8(43.3-121.1) | 0.8(0.4-1.4) | 90.7(40.9-172.3) | 0.5(0.2-1.1) | -1.90(-2.11--1.68) |
| Malawi | 27.7(12.6-48.4) | 0.3(0.2-0.5) | 22.9(12.7-43.0) | 0.2(0.1-0.4) | -1.99(-2.24--1.74) |
| Malaysia | 50.4(31.8-65.6) | 0.3(0.2-0.4) | 46.2(36.4-61.4) | 0.2(0.1-0.2) | -2.92(-3.19--2.65) |
| Maldives | 0.8(0.6-1.1) | 0.7(0.5-0.9) | 1.1(0.9-1.3) | 0.4(0.3-0.5) | -2.30(-2.48--2.13) |
| Mali | 50.7(27.9-83.8) | 1.0(0.5-1.7) | 49.9(29.1-78.0) | 0.5(0.3-0.8) | -3.45(-3.74--3.15) |
| Malta | 5.7(3.7-14.1) | 1.7(1.1-4.1) | 14.1(10.2-17.2) | 1.6(1.2-2.0) | -0.63(-1.60-0.35) |
| Marshall Islands | 0.6(0.4-0.0) | 2.9(2.1-4.0) | 1.1(0.8-1.5) | 3.4(2.4-4.4) | 0.47(0.03-0.90) |
| Mauritania | 8.6(5.3-11.1) | 0.8(0.5-1.2) | 8.0(5.5-11.7) | 0.4(0.3-0.6) | -3.19(-3.48--2.89) |
| Mauritius | 1.7(1.1-2.2) | 0.2(0.1-0.2) | 2.5(1.9-3.8) | 0.2(0.1-0.3) | -0.01(-0.37-0.36) |
| Mexico | 134.8(110.4-149.1) | 0.2(0.2-0.2) | 230.4(204.5-287.4) | 0.2(0.2-0.2) | 0.00(-0.19-0.20) |
| Moldova | 6.8(4.6-9.9) | 0.2(0.1-0.2) | 5.9(4.7-9.1) | 0.1(0.1-0.2) | -1.38(-1.94--0.82) |
| Mongolia | 26.7(17.5-36.3) | 2.2(1.3-3.1) | 46.6(30.6-60.1) | 2.0(1.2-2.6) | -0.85(-1.10--0.60) |
| Montenegro | 4.2(2.5-6.6) | 0.8(0.5-1.1) | 8.0(5.1-11.2) | 0.9(0.6-1.3) | 0.33(0.08-0.59) |
| Morocco | 75.5(56.4-100.1) | 0.3(0.3-0.4) | 87.6(66.5-119.7) | 0.3(0.2-0.4) | -0.71(-0.79--0.63) |
| Mozambique | 48.0(26.2-76.7) | 0.4(0.2-0.7) | 47.2(23.8-98.4) | 0.3(0.1-0.6) | -1.03(-1.38--0.68) |
| Myanmar | 176.2(94.2-349.3) | 0.5(0.3-0.8) | 202.7(141.6-294.3) | 0.5(0.3-0.7) | -0.34(-0.70-0.01) |
| Namibia | 5.4(3.5-7.7) | 0.6(0.4-0.9) | 6.3(3.3-9.7) | 0.4(0.2-0.6) | -2.19(-2.55--1.83) |
| Nepal | 38.8(20.4-67.6) | 0.3(0.2-0.5) | 61.3(36.0-92.6) | 0.3(0.2-0.4) | -0.12(-0.30-0.05) |
| Netherlands | 192.1(115.6-294.2) | 1.0(0.6-1.5) | 278.2(191.4-344.7) | 0.8(0.5-0.9) | -1.23(-2.05--0.41) |
| New Zealand | 18.8(16.0-26.2) | 0.5(0.5-0.8) | 23.8(20.0-28.4) | 0.4(0.4-0.5) | -0.96(-1.24--0.68) |
| Nicaragua | 5.4(3.5-7.7) | 0.2(0.1-0.2) | 5.9(4.8-7.3) | 0.1(0.1-0.1) | -2.53(-2.74--2.31) |
| Niger | 39.9(19.5-69.6) | 0.8(0.4-1.4) | 35.6(19.2-60.0) | 0.4(0.2-0.6) | -3.43(-3.77--3.08) |
| Nigeria | 406.8(257.2-590.5) | 0.8(0.5-1.2) | 343.7(222.0-505.6) | 0.3(0.2-0.5) | -4.07(-4.46--3.68) |
| North Korea | 101.5(79.1-125.1) | 0.7(0.5-0.8) | 214.8(153.2-281.7) | 0.9(0.6-1.2) | 1.29(0.83-1.75) |
| Northern Mariana Islands | 0.2(0.2-0.0) | 1.1(0.8-1.4) | 0.4(0.3-0.4) | 0.8(0.7-1.0) | -0.68(-1.09--0.26) |
| Norway | 45.0(35.7-49.4) | 0.7(0.6-0.7) | 69.3(55.9-75.9) | 0.6(0.5-0.7) | -0.63(-1.02--0.24) |
| Oman | 16.0(9.7-25.2) | 0.7(0.5-1.1) | 14.6(11.1-18.7) | 0.4(0.3-0.6) | -1.08(-1.98--0.16) |
| Pakistan | 484.4(259.3-767.7) | 0.7(0.4-1.2) | 1032.2(608.5-1791.5) | 0.8(0.5-1.5) | 0.26(0.09-0.43) |
| Palestine | 6.3(4.0-9.9) | 0.3(0.2-0.5) | 10.1(7.4-12.6) | 0.3(0.2-0.4) | 0.04(-0.14-0.22) |
| Panama | 2.7(1.8-3.3) | 0.1(0.1-0.2) | 5.7(4.5-8.2) | 0.1(0.1-0.2) | -0.36(-0.55--0.17) |
| Papua New Guinea | 59.3(39.0-97.9) | 2.6(1.7-4.2) | 131.3(90.6-195.4) | 2.5(1.7-3.7) | 0.06(-0.03-0.15) |
| Paraguay | 3.7(3.0-4.4) | 0.1(0.1-0.2) | 9.3(6.9-11.5) | 0.2(0.1-0.2) | 1.68(1.32-2.04) |
| Peru | 61.1(46.3-74.7) | 0.4(0.3-0.5) | 50.6(40.2-62.8) | 0.2(0.1-0.2) | -3.78(-3.99--3.57) |
| Philippines | 220.0(176.3-255.2) | 0.5(0.4-0.6) | 494.4(399.3-672.0) | 0.8(0.5-1.0) | 1.87(1.63-2.12) |
| Poland | 658.0(338.6-927.9) | 1.7(0.9-2.4) | 462.3(235.2-1141.1) | 0.7(0.4-1.6) | -4.86(-5.71--4.01) |
| Portugal | 30.7(18.1-83.8) | 0.3(0.2-0.8) | 113.6(75.9-145.8) | 0.4(0.3-0.5) | 1.51(-0.25-3.30) |
| Puerto Rico | 22.7(17.9-35.3) | 0.6(0.5-1.0) | 37.0(29.6-47.7) | 0.5(0.4-0.6) | -1.31(-1.79--0.83) |
| Qatar | 0.8(0.5-1.1) | 0.3(0.2-0.5) | 2.7(1.7-4.6) | 0.2(0.1-0.2) | -2.65(-2.84--2.46) |
| Romania | 1448.5(708.9-2170.2) | 6.7(3.6-9.7) | 1442.7(1076.9-1724.1) | 4.1(3.0-4.8) | -2.96(-3.45--2.46) |
| Russian Federation | 768.6(559.0-894.8) | 0.6(0.4-0.7) | 1439.1(1115.2-2193.6) | 0.7(0.5-1.0) | 0.52(0.18-0.85) |
| Rwanda | 27.5(15.6-44.4) | 0.6(0.3-1.1) | 20.8(8.4-40.9) | 0.3(0.1-0.6) | -3.52(-3.86--3.17) |
| Saint Lucia | 1.6(1.3-2.2) | 1.8(1.5-2.3) | 3.4(2.8-4.1) | 1.6(1.3-2.0) | -0.49(-0.62--0.36) |
| Saint Vincent and the Grenadines | 1.2(0.7-1.1) | 1.6(1.0-2.0) | 1.3(1.1-1.7) | 1.0(0.8-1.3) | -1.26(-1.53--1.00) |
| Samoa | 1.7(1.2-2.2) | 2.0(1.4-2.8) | 2.6(1.7-3.6) | 2.0(1.3-2.8) | 0.12(0.06-0.19) |
| Sao Tome and Principe | 0.5(0.4-0.0) | 0.6(0.4-0.8) | 0.5(0.4-0.7) | 0.4(0.3-0.5) | -2.18(-2.54--1.82) |
| Saudi Arabia | 92.9(60.0-133.1) | 0.7(0.5-0.9) | 124.7(96.6-161.4) | 0.6(0.5-0.8) | -0.10(-0.35-0.14) |
| Senegal | 23.4(14.1-33.3) | 0.6(0.3-0.8) | 25.4(17.8-34.6) | 0.3(0.2-0.5) | -2.68(-2.92--2.45) |
| Serbia | 106.8(72.8-134.1) | 1.1(0.8-1.4) | 114.0(58.8-269.8) | 0.9(0.5-1.9) | -0.95(-1.08--0.82) |
| Seychelles | 0.5(0.4-0.0) | 0.9(0.6-1.2) | 0.7(0.6-0.9) | 0.7(0.6-0.9) | -0.59(-0.74--0.45) |
| Sierra Leone | 18.4(11.8-26.2) | 0.7(0.5-1.0) | 18.2(13.5-23.5) | 0.4(0.3-0.5) | -2.12(-2.41--1.84) |
| Singapore | 62.9(37.2-78.7) | 2.0(1.4-2.4) | 46.5(36.6-66.8) | 0.7(0.6-1.0) | -4.07(-4.64--3.50) |
| Slovakia | 74.1(44.5-92.9) | 1.3(0.8-1.7) | 66.7(55.8-82.8) | 0.8(0.7-1.0) | -1.91(-2.16--1.66) |
| Slovenia | 11.2(3.1-42.4) | 0.6(0.2-2.1) | 73.9(27.4-111.7) | 1.5(0.6-2.2) | 4.92(4.04-5.80) |
| Solomon Islands | 2.3(1.4-3.3) | 1.5(0.9-2.4) | 5.3(3.4-7.7) | 1.7(1.1-2.4) | 0.34(0.19-0.49) |
| Somalia | 30.7(13.0-53.5) | 0.6(0.3-1.0) | 33.9(16.5-62.4) | 0.3(0.2-0.6) | -2.64(-2.99--2.28) |
| South Africa | 118.4(89.8-137.1) | 0.4(0.3-0.5) | 104.4(87.6-148.9) | 0.2(0.2-0.3) | -2.48(-3.21--1.75) |
| South Korea | 164.7(109.5-207.2) | 0.7(0.4-0.9) | 198.1(161.0-258.8) | 0.3(0.2-0.4) | -3.97(-5.09--2.85) |
| South Sudan | 21.2(8.2-37.3) | 0.5(0.2-1.0) | 21.4(9.9-40.9) | 0.3(0.2-0.6) | -2.25(-2.63--1.86) |
| Spain | 95.1(49.0-286.2) | 0.2(0.1-0.6) | 596.1(253.1-890.3) | 0.4(0.2-0.6) | 3.66(2.32-5.01) |
| Sri Lanka | 158.0(127.1-213.2) | 1.3(1.0-1.8) | 191.9(138.8-244.8) | 0.8(0.6-1.1) | -1.99(-2.44--1.54) |
| Sudan | 99.3(55.9-156.1) | 0.5(0.3-0.7) | 115.2(81.8-155.6) | 0.4(0.3-0.5) | -0.73(-0.79--0.67) |
| Suriname | 3.1(2.6-3.3) | 1.2(1.0-1.5) | 6.6(5.4-8.4) | 1.3(1.0-1.6) | 0.15(-0.03-0.33) |
| Swaziland | 2.8(2.0-3.3) | 0.6(0.4-0.8) | 3.3(2.3-4.8) | 0.4(0.3-0.6) | -0.76(-1.29--0.23) |
| Sweden | 48.4(17.9-123.1) | 0.4(0.1-0.9) | 172.7(128.6-203.9) | 0.8(0.6-1.0) | 3.29(1.62-4.99) |
| Switzerland | 53.2(36.4-73.7) | 0.5(0.4-0.7) | 70.0(54.4-102.2) | 0.3(0.3-0.5) | -2.17(-2.44--1.90) |
| Syria | 58.6(39.6-86.8) | 0.5(0.4-0.7) | 45.6(34.0-59.2) | 0.3(0.2-0.4) | -2.15(-2.49--1.81) |
| Taiwan (Province of China) | 39.5(33.7-53.5) | 0.2(0.2-0.3) | 138.8(76.4-175.8) | 0.4(0.2-0.5) | 3.95(3.33-4.58) |
| Tajikistan | 8.2(5.9-10.1) | 0.2(0.2-0.3) | 18.4(12.1-23.6) | 0.2(0.2-0.3) | 0.13(-0.06-0.32) |
| Tanzania | 86.9(46.9-147.1) | 0.5(0.2-0.8) | 106.3(48.5-200.6) | 0.3(0.1-0.6) | -2.10(-2.47--1.73) |
| Thailand | 223.1(144.7-275.2) | 0.6(0.3-0.7) | 205.8(176.0-253.3) | 0.3(0.2-0.3) | -3.93(-4.52--3.34) |
| The Bahamas | 1.1(0.9-1.1) | 0.7(0.6-0.9) | 2.2(1.8-2.7) | 0.7(0.5-0.8) | -0.45(-0.56--0.34) |
| The Gambia | 3.0(1.9-4.4) | 0.7(0.4-1.0) | 4.5(3.2-6.4) | 0.4(0.3-0.5) | -2.22(-2.47--1.97) |
| Timor-Leste | 5.3(2.7-8.8) | 0.7(0.4-1.0) | 5.7(3.1-9.7) | 0.6(0.3-1.1) | -0.37(-0.43--0.32) |
| Togo | 13.7(8.9-19.1) | 0.8(0.5-1.1) | 14.3(10.0-19.9) | 0.4(0.3-0.5) | -2.84(-3.19--2.49) |
| Tonga | 0.3(0.2-0.0) | 0.5(0.4-0.7) | 0.4(0.3-0.5) | 0.5(0.3-0.6) | -0.66(-0.78--0.54) |
| Trinidad and Tobago | 13.2(9.5-15.1) | 1.6(1.2-1.9) | 15.9(12.7-20.1) | 1.0(0.8-1.3) | -2.62(-3.10--2.14) |
| Tunisia | 24.4(17.5-32.3) | 0.4(0.2-0.5) | 27.4(17.8-39.5) | 0.2(0.2-0.4) | -1.37(-1.45--1.29) |
| Turkey | 96.4(69.7-131.1) | 0.2(0.1-0.3) | 109.1(86.3-130.9) | 0.1(0.1-0.2) | -0.99(-1.30--0.68) |
| Turkmenistan | 26.4(21.7-32.3) | 1.0(0.9-1.2) | 36.5(25.9-58.1) | 0.8(0.6-1.2) | -1.21(-2.13--0.28) |
| Uganda | 43.4(23.3-70.7) | 0.3(0.2-0.6) | 60.1(24.8-113.1) | 0.3(0.1-0.6) | -0.95(-1.11--0.79) |
| Ukraine | 205.6(154.2-309.3) | 0.4(0.3-0.6) | 522.8(400.2-740.9) | 0.8(0.6-1.0) | 2.61(2.02-3.19) |
| United Arab Emirates | 4.7(2.7-8.8) | 0.4(0.2-0.6) | 25.1(15.1-45.4) | 0.4(0.2-0.7) | 0.36(0.20-0.52) |
| United Kingdom | 339.7(310.9-432.4) | 0.4(0.4-0.5) | 830.9(573.0-947.4) | 0.6(0.4-0.7) | 2.23(1.37-3.10) |
| United States | 476.2(382.2-743.7) | 0.2(0.1-0.3) | 1146.6(780.0-1327.7) | 0.3(0.2-0.3) | 2.20(1.52-2.88) |
| Uruguay | 15.3(11.7-19.1) | 0.5(0.3-0.6) | 18.0(14.3-22.0) | 0.4(0.3-0.4) | -0.86(-1.09--0.63) |
| Uzbekistan | 65.1(47.6-83.8) | 0.4(0.3-0.6) | 212.7(175.0-301.7) | 0.8(0.6-1.1) | 2.70(2.47-2.93) |
| Vanuatu | 1.7(1.0-2.2) | 2.3(1.3-3.7) | 4.8(2.3-9.5) | 2.8(1.4-5.5) | 0.73(0.57-0.90) |
| Venezuela | 37.5(22.8-50.5) | 0.3(0.2-0.4) | 43.7(32.1-68.5) | 0.2(0.1-0.2) | -3.68(-4.34--3.02) |
| Vietnam | 214.2(145.3-298.2) | 0.4(0.3-0.6) | 368.3(252.2-474.8) | 0.4(0.3-0.5) | -0.41(-0.66--0.16) |
| Virgin Islands, U.S. | 0.7(0.6-0.0) | 1.0(0.8-1.3) | 2.2(1.7-2.6) | 1.4(1.1-1.7) | 1.65(1.27-2.04) |
| Yemen | 80.5(36.2-148.1) | 0.5(0.3-0.9) | 90.3(58.3-138.5) | 0.4(0.3-0.6) | -0.98(-1.14--0.83) |
| Zambia | 27.0(15.7-46.4) | 0.5(0.2-0.8) | 30.4(12.3-62.0) | 0.3(0.1-0.7) | -1.95(-2.17--1.73) |
| Zimbabwe | 35.4(21.4-67.6) | 0.6(0.3-1.2) | 73.9(44.3-137.6) | 0.7(0.4-1.5) | 1.66(0.93-2.40) |
