# Supplementary material for: Global, Regional, and National Burden of Myocarditis From 1990 to 2017: A Systematic Analysis Based on the Global Burden of Disease Study 2017
Source: Front Cardiovasc Med. 2021 Jul 2;8:692990. doi: 10.3389/fcvm.2021.692990 (PMC8284556; doi:10.3389/fcvm.2021.692990)
Supplement: Supplementary file 4 [file Table_4.docx]

**Table S4** The DALY and age-standardized DALY rate of myocarditis between 1990 and 2017, and its temporal trends from 1990 to 2017 in 195 countries and territories.

|  | 1990 |  | 2017 |  | 1990–2017 |
| --- | --- | --- | --- | --- | --- |
| Countries and territories | DALY No. (95% UI) | Age-standardized DALY Rate per 100,000 No. (95% UI) | DALY No. (95% UI) | Age-standardized DALY Rate per 100,000 No. (95% UI) | EAPC No. (95% CI) |
| Afghanistan | 1707.5(704.0-3015.9) | 17.2(6.7-32.0) | 3772.9(2550.9-5899.1) | 13.9(9.6-21.8) | -0.6(-0.4--0.8) |
| Albania | 4530.4(3671.7-5068.0) | 175.0(136.4-197.8) | 3263.3(2652.5-4023.4) | 96.5(78.8-120.3) | -2.6(-2.4--2.8) |
| Algeria | 5656.7(4018.2-7782.5) | 20.5(15.0-27.3) | 5596.0(3847.0-7679.7) | 13.7(9.5-19.1) | -1.2(-1.1--1.4) |
| American Samoa | 6.6(5.4-8.2) | 19.4(15.3-24.5) | 9.1(7.4-10.7) | 18.7(15.1-22.0) | 0.2(0.7--0.3) |
| Andorra | 9.0(6.4-12.2) | 17.9(12.9-24.1) | 14.3(10.7-18.3) | 13.9(10.6-17.8) | -0.8(-0.7--0.9) |
| Angola | 2714.7(1381.3-4325.7) | 23.3(12.8-36.1) | 3192.2(1958.8-5638.0) | 13.4(8.6-22.8) | -2.3(-2.1--2.5) |
| Antigua and Barbuda | 20.4(17.1-23.4) | 37.1(30.8-42.7) | 35.3(30.1-43.3) | 37.1(31.5-45.5) | 0.1(0.2--0.1) |
| Argentina | 7908.7(6314.1-9732.8) | 24.5(19.4-30.0) | 6061.1(5029.6-8056.3) | 13.2(10.8-18.0) | -2.6(-2.4--2.8) |
| Armenia | 182.2(114.1-231.4) | 5.7(3.4-7.3) | 164.1(119.0-283.3) | 4.9(3.7-8.0) | -0.7(-0.5--1.0) |
| Australia | 3151.1(2594.5-4465.7) | 19.2(15.8-27.3) | 3516.6(2953.7-4396.7) | 13.9(11.7-17.6) | -1.2(-0.8--1.7) |
| Austria | 3293.2(1659.9-6934.5) | 32.8(19.1-61.1) | 1933.6(1600.9-2758.7) | 13.7(11.4-17.9) | -3.5(-3.3--3.6) |
| Azerbaijan | 3374.9(2646.3-4731.1) | 50.3(39.2-72.2) | 4453.1(3379.5-6609.3) | 42.4(32.8-60.7) | -1.1(-0.6--1.5) |
| Bahrain | 42.6(31.4-56.2) | 9.0(6.6-11.5) | 57.6(44.2-71.6) | 4.2(3.4-5.1) | -3.9(-3.4--4.4) |
| Bangladesh | 25802.7(14983.8-41155.8) | 24.1(13.9-38.5) | 23350.2(14085.8-35614.8) | 15.6(9.5-23.7) | -1.2(-1.0--1.5) |
| Barbados | 61.0(50.4-71.1) | 21.8(18.1-25.2) | 76.3(64.1-91.3) | 19.2(16.3-22.8) | -0.6(-0.5--0.7) |
| Belarus | 1154.8(721.5-1752.7) | 10.2(6.4-15.5) | 870.9(521.6-1344.6) | 7.5(4.8-10.8) | -1.5(-1.1--2.0) |
| Belgium | 1279.8(936.2-2257.0) | 11.0(8.0-18.7) | 1981.1(1448.8-2388.2) | 10.6(8.0-12.4) | 0.2(0.7--0.3) |
| Belize | 45.7(34.2-58.0) | 28.7(22.1-34.6) | 98.4(82.4-127.0) | 30.5(25.6-39.1) | -0.1(0.3--0.4) |
| Benin | 1209.8(703.0-1833.7) | 24.4(14.3-37.3) | 1318.9(923.4-1899.9) | 13.1(9.2-18.7) | -2.5(-2.3--2.7) |
| Bermuda | 9.3(7.0-14.3) | 15.3(11.6-22.9) | 10.5(8.8-12.8) | 11.2(9.3-14.1) | -1.0(-0.8--1.1) |
| Bhutan | 125.0(80.2-190.5) | 24.2(15.6-36.5) | 130.4(84.1-213.3) | 15.4(10.0-25.3) | -2.0(-1.8--2.2) |
| Bolivia | 1522.6(931.7-2219.9) | 24.3(15.0-34.6) | 1132.6(813.9-1573.9) | 10.8(7.9-14.8) | -3.1(-3.1--3.2) |
| Bosnia and Herzegovina | 1360.3(841.1-1910.1) | 33.5(20.6-47.1) | 2084.1(1212.4-2868.3) | 42.7(25.0-58.0) | 0.8(1.0-0.7) |
| Botswana | 205.0(144.6-272.0) | 17.9(12.8-23.5) | 242.4(161.2-345.3) | 11.7(7.9-16.2) | -1.6(-1.4--1.8) |
| Brazil | 21077.1(17349.9-30384.3) | 14.3(12.0-20.7) | 22075.9(18184.9-32233.6) | 11.1(9.1-16.2) | -1.0(-0.7--1.3) |
| Brunei | 123.8(75.2-163.1) | 48.2(32.0-60.4) | 161.7(127.6-194.1) | 36.8(29.6-44.0) | -1.1(-0.5--1.6) |
| Bulgaria | 3492.5(2651.9-5090.2) | 35.9(28.6-50.8) | 5159.2(4530.6-6142.6) | 49.2(43.4-59.7) | 0.7(1.3-0.0) |
| Burkina Faso | 2383.0(1358.6-3770.3) | 25.2(15.7-39.0) | 2658.7(1794.1-3774.6) | 14.1(10.0-19.9) | -2.4(-2.1--2.7) |
| Burundi | 1455.7(803.0-2544.5) | 24.7(13.6-43.3) | 1139.0(552.7-2136.5) | 11.1(5.4-21.5) | -3.4(-3.1--3.6) |
| Cambodia | 2829.8(1455.1-5329.6) | 23.8(13.8-36.9) | 2872.4(2122.3-3820.7) | 19.4(14.3-25.7) | -1.0(-0.7--1.2) |
| Cameroon | 2994.5(1984.4-4118.5) | 30.8(21.2-41.6) | 3749.4(2860.1-4986.2) | 15.9(12.2-20.9) | -2.8(-2.6--3.0) |
| Canada | 2074.9(1539.6-3108.0) | 8.1(5.9-12.5) | 3807.1(2504.5-4731.5) | 11.0(7.6-13.4) | 1.5(2.0-1.1) |
| Cape Verde | 39.7(28.9-52.5) | 11.8(8.7-15.3) | 54.5(45.1-65.7) | 9.9(8.2-11.9) | -0.7(-0.6--0.8) |
| Central African Republic | 651.0(332.6-1092.2) | 23.3(12.4-37.3) | 802.2(434.0-1532.7) | 18.9(10.7-34.2) | -1.0(-0.8--1.1) |
| Chad | 1530.2(827.3-2638.0) | 23.4(13.2-38.7) | 2007.5(1321.5-3006.0) | 14.0(9.6-20.9) | -2.0(-1.9--2.2) |
| Chile | 769.7(640.9-931.0) | 5.8(5.0-7.1) | 963.1(814.5-1167.7) | 5.2(4.4-6.2) | -0.3(0.1--0.6) |
| China | 394090.7(315487.8-456761.9) | 34.4(27.7-39.7) | 396722.8(338878.3-430790.7) | 29.3(25.4-31.6) | -0.3(0.1--0.7) |
| Colombia | 4718.6(3273.6-5684.7) | 13.7(9.9-16.2) | 5122.7(3986.7-6363.9) | 10.5(8.1-13.0) | -1.2(-0.9--1.5) |
| Comoros | 107.3(70.2-166.5) | 22.7(14.5-34.8) | 66.2(30.8-117.5) | 10.3(4.7-18.6) | -3.2(-2.9--3.5) |
| Congo | 484.7(324.2-772.1) | 22.3(15.2-34.4) | 651.4(366.6-1192.2) | 15.7(8.6-28.2) | -1.8(-1.5--2.0) |
| Costa Rica | 503.7(314.9-641.6) | 16.5(10.9-20.5) | 674.9(509.6-814.6) | 14.1(10.6-17.1) | -0.9(-0.5--1.3) |
| Cote d'Ivoire | 2754.7(1784.2-3734.9) | 23.7(15.3-32.0) | 3531.0(2583.1-4921.8) | 15.9(11.6-22.3) | -1.8(-1.6--2.0) |
| Croatia | 2512.2(2160.7-3168.6) | 45.3(39.4-58.7) | 3183.7(2533.4-3716.8) | 40.3(35.0-46.8) | -1.5(0.6--3.7) |
| Cuba | 1667.2(1380.7-2113.6) | 16.2(13.3-20.5) | 1915.3(1507.9-2943.5) | 13.4(11.0-19.2) | -0.8(-0.5--1.1) |
| Cyprus | 203.8(157.6-246.1) | 26.9(20.7-32.7) | 241.4(187.7-283.8) | 15.3(12.6-17.7) | -2.2(-1.9--2.6) |
| Czech Republic | 2025.6(1382.2-3842.2) | 18.5(12.8-34.5) | 3565.8(2707.3-4202.2) | 23.1(17.5-27.1) | 0.4(0.8--0.1) |
| Democratic Republic of the Congo | 7501.5(4359.2-11843.6) | 18.1(11.2-26.6) | 9349.8(5246.8-16000.4) | 12.7(7.2-20.1) | -1.5(-1.4--1.6) |
| Denmark | 1325.2(963.1-1562.2) | 20.9(15.9-24.1) | 1038.6(892.7-1194.1) | 12.3(10.7-14.3) | -2.3(-2.1--2.5) |
| Djibouti | 110.5(62.5-190.6) | 22.9(11.8-39.9) | 117.7(52.3-238.5) | 11.9(5.2-24.6) | -3.0(-2.8--3.3) |
| Dominica | 19.2(13.9-23.5) | 26.5(19.3-32.0) | 22.1(18.2-28.2) | 27.4(22.8-35.4) | 0.0(0.2--0.1) |
| Dominican Republic | 1547.0(1250.8-1939.1) | 26.0(20.1-31.3) | 1697.2(1368.8-2137.8) | 17.6(14.1-21.9) | -1.5(-1.2--1.9) |
| Ecuador | 1115.2(701.8-1414.0) | 12.3(7.7-15.6) | 1293.2(1020.4-1796.6) | 7.9(6.2-11.0) | -2.4(-2.1--2.7) |
| Egypt | 19959.4(11542.3-29074.7) | 30.7(19.3-42.7) | 12762.6(9124.3-18293.8) | 14.0(10.0-20.2) | -3.1(-3.0--3.3) |
| El Salvador | 651.2(548.9-775.5) | 15.0(12.1-18.2) | 811.0(571.9-1039.1) | 13.7(9.6-17.7) | -0.1(0.1--0.3) |
| Equatorial Guinea | 108.3(58.8-169.6) | 23.0(13.0-34.3) | 113.0(57.0-232.3) | 11.2(6.0-23.0) | -3.1(-2.9--3.4) |
| Eritrea | 909.4(472.4-1617.2) | 33.1(16.1-58.0) | 947.5(323.3-1732.9) | 19.2(6.4-36.7) | -2.7(-2.4--3.0) |
| Estonia | 89.8(57.8-113.6) | 5.4(3.4-7.0) | 91.6(63.9-114.5) | 5.5(3.2-7.3) | 0.1(0.5--0.3) |
| Ethiopia | 13519.3(8103.5-22001.3) | 21.7(13.0-34.2) | 9040.2(4970.4-14819.8) | 10.2(5.2-16.9) | -3.1(-3.0--3.3) |
| Federated States of Micronesia | 58.9(40.3-83.5) | 80.0(55.2-113.4) | 58.9(39.6-84.5) | 68.6(47.1-96.9) | -0.7(-0.5--0.8) |
| Fiji | 704.1(591.0-823.4) | 130.5(108.5-154.3) | 846.8(688.4-1070.3) | 103.1(84.8-130.6) | -1.2(-0.8--1.6) |
| Finland | 2326.3(1477.5-2931.7) | 41.9(27.5-52.2) | 1660.0(1424.1-1975.5) | 23.1(19.7-26.9) | -2.3(-2.0--2.5) |
| France | 3214.6(2264.8-5559.6) | 5.3(4.0-8.2) | 5962.3(3858.6-7486.1) | 6.9(4.0-8.8) | 1.8(2.4-1.3) |
| Gabon | 172.4(117.9-278.8) | 19.3(13.5-31.6) | 204.1(126.1-388.4) | 13.8(8.5-26.4) | -1.3(-1.1--1.4) |
| Georgia | 2322.5(1574.4-2936.8) | 40.1(27.6-50.8) | 2213.5(818.0-3132.4) | 46.5(18.9-64.8) | 0.5(1.0-0.1) |
| Germany | 21204.1(17390.7-26988.2) | 22.5(18.8-29.1) | 19773.9(16427.4-26755.2) | 15.8(13.4-20.2) | -1.6(-1.3--2.0) |
| Ghana | 3329.1(1758.3-4806.3) | 24.4(13.2-34.9) | 4219.1(3185.3-5604.0) | 16.1(12.5-21.2) | -2.0(-1.6--2.4) |
| Greece | 2303.5(919.6-3221.3) | 18.2(8.0-25.1) | 1330.8(1021.9-1603.3) | 7.6(6.1-8.9) | -2.9(-1.2--4.5) |
| Greenland | 27.8(21.8-36.4) | 52.0(40.9-66.7) | 16.0(11.0-20.2) | 30.9(21.5-40.0) | -1.8(-1.5--2.1) |
| Grenada | 50.5(38.4-60.8) | 65.9(49.7-79.4) | 55.9(47.7-69.3) | 39.7(34.2-49.4) | -1.9(-1.6--2.1) |
| Guam | 36.0(29.1-44.4) | 33.3(26.9-41.2) | 56.4(47.3-65.1) | 32.3(27.2-37.3) | 0.1(0.5--0.2) |
| Guatemala | 965.2(745.4-1381.1) | 12.8(9.7-15.5) | 1959.8(1645.4-2561.2) | 12.6(10.6-15.4) | -0.3(-0.2--0.5) |
| Guinea | 2098.3(1145.6-3414.1) | 30.1(16.5-47.4) | 1587.2(1076.0-2256.8) | 14.9(10.1-21.3) | -2.8(-2.5--3.1) |
| Guinea-Bissau | 338.0(212.2-506.8) | 35.6(23.0-51.6) | 270.2(199.5-363.2) | 17.0(12.5-22.7) | -2.9(-2.7--3.1) |
| Guyana | 332.1(243.6-513.5) | 52.9(39.8-79.1) | 518.8(406.7-618.0) | 77.4(61.7-91.4) | 1.6(2.0-1.2) |
| Haiti | 4771.1(2769.3-7607.2) | 73.0(45.6-114.4) | 4652.9(3079.6-6724.5) | 48.1(31.8-69.7) | -1.5(-1.4--1.6) |
| Honduras | 955.3(717.4-1198.4) | 24.6(18.0-31.6) | 1112.2(745.5-1643.4) | 14.2(9.7-21.0) | -1.9(-1.6--2.2) |
| Hungary | 2428.2(1908.1-3341.1) | 21.0(16.0-27.8) | 2781.3(1842.0-3344.3) | 18.9(12.5-22.5) | 0.9(1.6-0.2) |
| Iceland | 31.9(23.9-49.5) | 12.1(9.1-18.6) | 43.6(37.7-50.4) | 10.2(8.8-12.1) | -0.5(-0.1--1.0) |
| India | 147575.1(98426.7-233694.3) | 18.2(12.0-28.7) | 210638.8(138815.5-300881.9) | 16.6(10.9-23.7) | -0.5(-0.3--0.7) |
| Indonesia | 35703.0(25973.7-45129.9) | 19.8(14.4-25.2) | 39815.3(28432.9-59088.8) | 16.3(11.7-24.2) | -0.7(-0.6--0.8) |
| Iran | 11961.8(8698.4-14686.1) | 19.3(14.7-23.3) | 10451.6(9024.7-12868.5) | 13.5(11.7-16.6) | -0.4(0.0--0.8) |
| Iraq | 19652.2(12367.1-27867.7) | 87.0(59.9-116.9) | 22191.4(17138.8-27934.7) | 45.0(36.3-55.2) | -2.3(-2.0--2.7) |
| Ireland | 439.0(329.5-742.8) | 12.3(9.4-20.4) | 996.3(464.6-1367.9) | 16.1(7.7-21.8) | 2.0(2.5-1.4) |
| Israel | 699.6(588.6-852.6) | 14.4(12.2-17.5) | 863.4(723.8-1037.8) | 9.0(7.4-10.8) | -2.1(-1.6--2.5) |
| Italy | 4790.6(2671.2-12148.3) | 7.1(4.2-16.2) | 12959.1(5379.1-18439.0) | 9.9(4.7-13.0) | 3.4(5.5-1.5) |
| Jamaica | 402.2(297.2-483.5) | 17.9(13.1-21.4) | 424.4(343.7-554.8) | 15.3(12.4-19.9) | -1.2(-0.9--1.6) |
| Japan | 40124.7(32950.6-44650.9) | 29.9(24.7-33.0) | 28307.7(22878.1-37782.6) | 15.6(12.7-20.3) | -2.5(-2.4--2.6) |
| Jordan | 658.1(490.2-841.8) | 17.1(13.3-21.0) | 739.0(617.5-881.7) | 7.5(6.3-8.8) | -3.8(-3.3--4.3) |
| Kazakhstan | 1006.6(714.4-2073.7) | 6.3(4.5-12.8) | 8915.0(7045.7-12653.1) | 49.0(39.2-67.1) | 11.0(13.0-9.0) |
| Kenya | 2936.7(1938.3-4585.0) | 13.9(8.1-22.7) | 3985.1(1916.0-7084.4) | 10.4(4.7-19.5) | -1.3(-1.2--1.5) |
| Kiribati | 78.4(62.3-111.1) | 130.9(105.5-180.4) | 119.9(88.5-177.9) | 123.0(92.3-180.1) | -0.1(0.0--0.2) |
| Kuwait | 409.0(347.9-474.7) | 21.3(18.2-24.7) | 560.1(474.6-649.1) | 12.2(10.4-14.1) | -1.8(-1.5--2.1) |
| Kyrgyzstan | 751.4(478.1-979.2) | 18.4(11.1-25.1) | 976.8(646.6-1251.9) | 15.7(10.2-20.2) | -1.0(-0.7--1.4) |
| Laos | 2923.4(1342.1-4718.7) | 52.8(29.4-80.8) | 2326.2(1614.2-3179.6) | 33.7(23.8-46.6) | -1.7(-1.4--2.0) |
| Latvia | 141.4(85.9-180.9) | 5.1(2.9-6.7) | 93.1(75.5-129.5) | 4.1(2.7-5.1) | -1.3(-0.8--1.8) |
| Lebanon | 533.5(424.1-670.1) | 12.9(10.4-16.0) | 579.2(432.6-810.4) | 7.1(5.3-9.9) | -2.8(-2.5--3.0) |
| Lesotho | 320.9(204.9-477.0) | 19.4(12.4-28.6) | 347.3(230.7-497.4) | 19.4(13.1-27.5) | 0.6(0.8-0.3) |
| Liberia | 580.7(351.3-898.4) | 27.7(17.7-39.5) | 462.8(323.4-618.1) | 11.1(7.8-14.7) | -3.6(-3.2--4.1) |
| Libya | 3169.0(2132.1-4440.2) | 56.0(38.6-77.7) | 1393.4(873.3-2174.9) | 21.3(13.1-33.5) | -3.8(-3.6--4.1) |
| Lithuania | 179.8(140.9-217.0) | 4.8(3.6-5.8) | 175.8(140.2-229.5) | 5.2(3.6-6.4) | 0.2(0.5--0.2) |
| Luxembourg | 93.5(70.4-152.6) | 21.4(16.3-34.1) | 156.1(120.2-186.5) | 17.9(13.7-21.2) | -0.6(-0.1--1.2) |
| Macedonia | 314.7(150.7-455.2) | 17.1(8.3-25.2) | 319.4(180.8-475.6) | 13.2(7.0-19.5) | -0.5(-0.1--1.0) |
| Madagascar | 4527.8(2787.5-7297.9) | 36.8(21.2-59.1) | 4947.7(2495.3-8936.3) | 21.6(10.2-39.8) | -2.4(-2.1--2.6) |
| Malawi | 1988.7(899.0-3509.5) | 14.9(7.2-25.0) | 1339.6(781.3-2423.3) | 8.5(5.0-15.1) | -2.5(-2.2--2.8) |
| Malaysia | 3326.0(2057.9-4436.9) | 17.6(11.4-22.6) | 2702.1(2208.9-3356.1) | 9.1(7.4-11.2) | -2.9(-2.6--3.1) |
| Maldives | 50.8(32.3-73.1) | 22.3(16.2-30.2) | 41.0(34.2-49.0) | 10.6(9.0-12.3) | -2.8(-2.7--3.0) |
| Mali | 3163.9(1734.9-5208.6) | 35.2(20.0-57.7) | 3026.4(1818.6-4568.3) | 15.6(9.6-23.8) | -3.6(-3.3--3.9) |
| Malta | 141.3(103.5-250.6) | 38.1(28.1-67.3) | 218.1(172.8-252.7) | 34.5(27.4-39.5) | -0.5(0.1--1.2) |
| Marshall Islands | 23.1(16.4-30.9) | 80.8(57.6-108.2) | 43.4(30.5-57.4) | 96.4(68.3-126.8) | 0.6(1.1-0.1) |
| Mauritania | 434.0(278.5-602.8) | 23.8(15.1-32.6) | 374.5(267.3-525.0) | 11.3(8.1-16.2) | -3.0(-2.8--3.3) |
| Mauritius | 93.6(66.6-111.4) | 8.8(6.3-10.5) | 104.8(83.0-151.3) | 8.1(6.5-11.6) | -0.2(0.1--0.5) |
| Mexico | 8080.3(6485.8-8923.6) | 9.5(7.7-10.5) | 10521.1(9190.4-13441.3) | 8.5(7.4-10.8) | -0.1(0.1--0.3) |
| Moldova | 298.2(173.9-379.9) | 6.7(4.1-8.5) | 218.6(159.5-271.9) | 5.4(3.9-6.5) | -1.0(-0.5--1.5) |
| Mongolia | 1119.3(832.2-1429.1) | 68.9(47.5-93.0) | 1762.7(1276.9-2242.1) | 57.5(41.0-72.4) | -1.0(-0.8--1.3) |
| Montenegro | 110.4(75.4-142.4) | 18.9(13.0-24.2) | 133.5(93.3-171.0) | 16.3(11.8-20.4) | -0.9(-0.7--1.1) |
| Morocco | 4781.2(3442.2-6696.2) | 17.6(13.1-23.2) | 4051.7(3091.4-5448.9) | 11.9(9.1-16.2) | -1.3(-1.3--1.4) |
| Mozambique | 3355.7(1732.8-5302.4) | 18.0(10.4-28.6) | 2624.4(1375.9-5156.9) | 10.9(5.7-22.1) | -2.0(-1.6--2.4) |
| Myanmar | 11579.5(5530.8-26669.7) | 26.4(13.9-55.4) | 9416.6(6695.5-12426.6) | 19.8(14.0-26.8) | -1.2(-0.9--1.6) |
| Namibia | 283.8(189.5-397.1) | 21.8(14.5-31.0) | 311.9(168.5-464.6) | 14.2(7.8-21.2) | -1.9(-1.5--2.2) |
| Nepal | 2185.6(1299.9-3690.5) | 12.0(6.8-20.2) | 2468.3(1600.6-3540.9) | 9.4(6.0-13.5) | -0.8(-0.6--1.0) |
| Netherlands | 3623.4(2733.5-4457.9) | 21.5(16.9-26.4) | 3394.3(2747.7-3951.8) | 13.0(10.9-15.8) | -2.0(-1.5--2.5) |
| New Zealand | 984.1(845.6-1293.4) | 29.3(25.2-38.3) | 946.7(821.5-1077.5) | 20.3(17.6-22.9) | -1.3(-1.1--1.6) |
| Nicaragua | 333.1(212.0-453.8) | 8.7(5.8-11.2) | 292.1(237.6-347.5) | 4.8(4.0-5.8) | -2.5(-2.3--2.7) |
| Niger | 2753.8(1336.6-4860.0) | 29.3(15.3-49.5) | 2233.1(1285.7-3647.8) | 11.7(6.8-19.3) | -3.8(-3.5--4.1) |
| Nigeria | 21550.7(13686.8-31495.8) | 24.1(15.5-34.6) | 18522.3(12896.4-26002.8) | 10.3(6.8-14.7) | -3.9(-3.5--4.3) |
| North Korea | 4852.3(3779.4-6050.3) | 24.8(19.5-30.7) | 7617.7(5520.9-9847.3) | 33.5(24.2-43.3) | 1.3(1.8-0.9) |
| Northern Mariana Islands | 9.0(6.8-11.7) | 28.1(22.0-35.9) | 10.9(9.1-13.1) | 21.5(18.1-25.2) | -0.6(-0.3--1.0) |
| Norway | 905.6(809.4-1024.7) | 17.9(16.2-21.1) | 965.4(848.0-1067.5) | 12.8(11.4-14.5) | -1.3(-1.1--1.6) |
| Oman | 1279.4(735.1-2091.4) | 49.3(30.4-77.1) | 981.0(747.9-1247.3) | 24.4(18.7-31.0) | -2.0(-0.8--3.1) |
| Pakistan | 21044.7(12851.5-31092.0) | 24.0(13.4-37.3) | 45681.5(29252.4-73238.0) | 25.4(15.7-42.5) | -0.1(0.1--0.4) |
| Palestine | 441.6(265.5-664.4) | 16.5(10.6-23.8) | 532.0(416.5-661.7) | 12.0(9.3-14.6) | -1.1(-0.9--1.3) |
| Panama | 153.8(101.3-194.7) | 6.6(4.5-8.2) | 247.2(205.8-307.6) | 6.4(5.3-7.9) | -0.1(0.0--0.3) |
| Papua New Guinea | 2832.8(1894.6-4695.3) | 85.5(56.6-138.7) | 6054.5(4134.3-9006.5) | 80.1(55.9-116.9) | 0.0(0.1--0.1) |
| Paraguay | 228.4(187.4-282.7) | 5.7(4.7-6.9) | 407.7(311.8-503.3) | 6.2(4.8-7.6) | 0.9(1.3-0.6) |
| Peru | 3168.3(2375.0-4011.0) | 14.4(11.2-17.5) | 1923.3(1562.4-2323.8) | 5.9(4.8-7.1) | -3.6(-3.5--3.7) |
| Philippines | 13215.9(10985.2-15566.7) | 20.1(16.6-23.4) | 23068.0(18500.2-31745.9) | 23.7(19.4-32.0) | 0.6(0.8-0.3) |
| Poland | 13810.5(8171.7-17788.0) | 34.1(20.3-43.6) | 8540.8(5950.4-15441.7) | 15.9(11.8-25.9) | -3.7(-3.1--4.3) |
| Portugal | 906.8(630.8-1761.5) | 8.8(6.1-16.8) | 1404.4(1096.7-1658.7) | 7.5(6.2-8.8) | -0.6(0.4--1.7) |
| Puerto Rico | 635.5(537.7-847.2) | 17.9(15.2-23.6) | 685.4(582.6-839.6) | 14.3(12.3-17.1) | -1.1(-0.7--1.6) |
| Qatar | 58.6(37.2-98.6) | 13.7(9.2-22.1) | 184.7(125.6-304.7) | 7.6(5.4-11.2) | -2.4(-2.2--2.5) |
| Romania | 27401.1(14503.8-41299.5) | 109.8(62.3-158.6) | 26789.1(18158.6-33466.1) | 86.2(59.5-107.0) | -1.9(-1.4--2.3) |
| Russian Federation | 18323.7(15111.0-23381.5) | 12.7(10.4-15.9) | 28043.7(22217.9-36104.3) | 15.9(13.0-19.7) | 0.8(1.1-0.4) |
| Rwanda | 1640.7(984.0-2574.0) | 22.6(13.1-36.7) | 1098.9(532.0-1966.7) | 10.1(4.7-18.8) | -4.1(-3.6--4.5) |
| Saint Lucia | 40.3(33.4-50.7) | 37.8(31.1-47.4) | 69.6(58.7-84.1) | 35.2(29.8-42.3) | -0.3(-0.2--0.4) |
| Saint Vincent and the Grenadines | 35.4(22.6-43.8) | 38.4(23.9-47.7) | 34.9(29.7-43.5) | 28.6(24.3-35.9) | -0.8(-0.6--1.1) |
| Samoa | 63.1(46.3-86.4) | 53.7(39.0-74.5) | 79.8(54.9-110.4) | 50.2(34.2-70.0) | -0.3(-0.2--0.3) |
| Sao Tome and Principe | 29.0(20.1-38.7) | 23.6(16.4-31.9) | 27.8(19.8-38.7) | 14.7(10.7-19.9) | -2.2(-1.9--2.6) |
| Saudi Arabia | 6343.0(3862.5-9433.0) | 31.9(21.4-44.6) | 6272.6(4980.5-7884.7) | 19.9(16.2-24.7) | -1.5(-1.3--1.7) |
| Senegal | 1356.1(838.3-1909.5) | 18.5(11.6-25.9) | 1304.6(975.7-1694.8) | 10.4(7.6-13.8) | -2.5(-2.3--2.7) |
| Serbia | 3955.5(2247.4-5378.2) | 44.4(23.9-61.4) | 2307.6(1592.0-3776.3) | 20.4(15.3-29.0) | -3.0(-2.7--3.2) |
| Seychelles | 17.6(13.2-22.8) | 25.9(19.2-33.9) | 23.4(19.7-27.5) | 23.1(19.6-27.2) | -0.5(-0.3--0.6) |
| Sierra Leone | 1149.5(700.4-1721.4) | 26.1(16.9-37.1) | 1025.3(767.1-1340.7) | 14.1(10.8-18.1) | -2.4(-2.1--2.6) |
| Singapore | 3412.2(1884.9-4343.5) | 101.3(60.9-126.7) | 2224.2(1913.7-2603.3) | 36.0(30.9-41.9) | -3.9(-3.3--4.5) |
| Slovakia | 2195.6(1658.2-2589.4) | 40.0(31.1-46.8) | 1533.8(1253.9-2023.4) | 21.5(17.2-30.1) | -2.4(-2.1--2.6) |
| Slovenia | 261.8(127.1-671.7) | 12.7(6.7-30.9) | 778.6(393.9-1078.6) | 20.1(10.9-26.7) | 2.5(3.2-1.7) |
| Solomon Islands | 91.5(58.6-144.9) | 42.7(27.4-67.7) | 190.7(126.0-279.5) | 43.3(28.4-63.1) | 0.2(0.3-0.0) |
| Somalia | 2043.2(852.2-3636.4) | 24.6(11.0-42.4) | 2074.4(1055.7-3725.0) | 13.2(6.9-23.3) | -3.1(-2.7--3.4) |
| South Africa | 6645.4(4929.8-7757.3) | 18.1(13.8-20.8) | 4977.2(4071.8-7109.5) | 9.2(7.6-13.0) | -2.8(-2.0--3.5) |
| South Korea | 7304.3(5160.8-8696.2) | 19.7(13.4-23.5) | 5341.3(4494.9-6451.9) | 9.3(7.8-10.9) | -3.1(-2.5--3.6) |
| South Sudan | 1331.8(532.4-2381.6) | 21.0(8.7-36.8) | 1373.8(620.1-2516.3) | 13.3(6.8-24.3) | -2.1(-1.6--2.5) |
| Spain | 2797.4(1989.7-5198.2) | 7.1(5.3-11.9) | 5969.0(3655.7-7702.0) | 8.1(4.9-10.0) | 1.0(1.8-0.3) |
| Sri Lanka | 7280.6(5941.5-9494.6) | 46.5(37.8-61.5) | 7110.1(5139.5-8945.4) | 31.8(23.0-40.0) | -1.6(-1.1--2.1) |
| Sudan | 7284.5(3643.7-12096.5) | 25.4(14.9-39.2) | 7477.9(5175.3-10714.5) | 16.8(12.2-22.7) | -1.3(-1.2--1.4) |
| Suriname | 108.3(86.7-141.9) | 32.2(26.8-41.0) | 170.1(145.6-208.1) | 30.5(26.1-37.4) | -0.2(0.1--0.4) |
| Swaziland | 164.7(113.5-218.3) | 22.3(16.0-28.2) | 181.9(125.4-257.3) | 17.5(12.2-24.7) | -0.4(0.1--1.0) |
| Sweden | 1545.8(860.5-2953.0) | 15.4(8.8-29.0) | 3155.8(2152.1-3881.8) | 23.0(15.2-28.6) | 1.8(2.9-0.6) |
| Switzerland | 1231.4(887.9-1479.5) | 15.6(11.0-18.5) | 1021.0(849.2-1293.5) | 8.3(7.0-10.3) | -2.8(-2.6--3.0) |
| Syria | 4198.9(2677.7-6358.8) | 27.6(19.2-40.1) | 2298.0(1703.9-3009.5) | 14.0(10.4-18.3) | -3.2(-2.8--3.6) |
| Taiwan (Province of China) | 2486.2(2122.5-3125.8) | 12.3(10.5-15.7) | 4804.3(3091.9-5892.8) | 18.8(12.5-22.6) | 2.7(3.1-2.3) |
| Tajikistan | 448.1(319.4-551.2) | 10.2(7.2-12.6) | 957.5(623.3-1225.3) | 10.9(7.3-13.9) | 0.1(0.3--0.1) |
| Tanzania | 5576.5(3005.5-9449.7) | 18.6(10.3-31.5) | 6155.1(3271.2-11004.8) | 11.7(5.8-21.0) | -2.1(-1.8--2.4) |
| Thailand | 11071.3(7809.3-13381.0) | 21.5(14.8-25.9) | 8118.0(6777.6-10233.5) | 11.5(9.5-14.8) | -3.3(-2.6--4.0) |
| The Bahamas | 38.0(30.9-46.4) | 18.1(14.6-21.8) | 60.5(49.1-73.6) | 16.3(13.3-19.9) | -0.3(-0.3--0.4) |
| The Gambia | 180.8(116.1-259.1) | 20.2(13.1-29.6) | 233.8(171.4-342.5) | 12.9(9.5-18.4) | -1.8(-1.6--2.0) |
| Timor-Leste | 396.1(177.7-637.2) | 36.1(19.6-54.7) | 280.0(162.0-430.3) | 22.9(12.9-37.2) | -2.0(-1.9--2.2) |
| Togo | 832.0(544.1-1198.6) | 23.8(15.8-33.5) | 771.8(568.6-1051.5) | 12.1(8.9-16.3) | -2.8(-2.5--3.1) |
| Tonga | 9.8(7.2-12.8) | 13.5(10.0-17.7) | 11.9(9.2-15.5) | 13.1(10.2-17.1) | -0.2(-0.1--0.3) |
| Trinidad and Tobago | 458.6(339.7-537.1) | 42.3(31.7-49.3) | 447.9(359.4-556.3) | 30.9(25.1-38.1) | -1.9(-1.5--2.2) |
| Tunisia | 1557.2(1101.7-2206.4) | 17.4(12.7-23.8) | 1003.2(669.6-1361.3) | 8.8(5.8-12.2) | -2.5(-2.3--2.6) |
| Turkey | 6227.1(4136.3-8977.7) | 10.3(7.2-14.4) | 5316.6(4338.2-6904.6) | 7.2(5.8-9.4) | -0.8(-0.4--1.1) |
| Turkmenistan | 1353.4(980.9-1674.3) | 39.3(33.4-50.0) | 1653.4(1210.9-2602.5) | 33.6(25.2-51.4) | -1.1(-0.3--1.9) |
| Uganda | 2904.2(1458.0-4597.8) | 14.1(8.2-23.0) | 3520.3(1726.7-6259.2) | 10.6(4.6-19.2) | -1.3(-1.1--1.4) |
| Ukraine | 4863.2(4008.0-6400.4) | 8.8(7.3-11.3) | 9110.4(7483.1-11794.2) | 17.0(13.8-21.7) | 2.1(2.7-1.5) |
| United Arab Emirates | 296.2(189.5-488.3) | 15.8(10.1-24.7) | 1359.0(911.5-2229.9) | 14.7(10.3-22.1) | 0.1(0.2--0.1) |
| United Kingdom | 8717.3(7795.5-10826.5) | 13.9(12.5-17.3) | 13356.7(9994.1-15065.5) | 15.5(11.8-17.4) | 1.0(1.6-0.3) |
| United States | 27369.1(22430.2-39185.8) | 11.6(9.5-16.5) | 47655.4(35108.3-54370.4) | 15.9(11.9-18.0) | 1.5(2.0-0.9) |
| Uruguay | 625.3(480.2-743.7) | 20.2(15.4-24.0) | 526.8(408.6-636.7) | 14.2(11.0-17.1) | -1.5(-1.2--1.7) |
| Uzbekistan | 3559.4(2686.5-4271.7) | 19.1(14.1-24.5) | 10093.5(8378.5-13114.1) | 31.5(26.1-41.7) | 2.4(2.6-2.1) |
| Vanuatu | 68.7(39.9-109.5) | 67.3(37.6-109.7) | 170.8(89.4-328.2) | 78.2(39.7-154.5) | 0.6(0.7-0.4) |
| Venezuela | 1970.0(1217.0-2564.9) | 11.2(6.9-14.8) | 2018.1(1597.5-2804.4) | 6.6(5.2-9.1) | -3.1(-2.6--3.7) |
| Vietnam | 11027.5(7747.1-14966.5) | 17.4(12.3-23.7) | 13640.6(9577.9-17692.3) | 14.4(10.2-18.6) | -0.9(-0.7--1.0) |
| Virgin Islands, U.S. | 21.7(18.2-27.9) | 23.2(19.5-29.1) | 45.0(35.2-55.4) | 31.3(24.8-38.1) | 1.4(1.8-1.0) |
| Yemen | 6187.5(2592.3-11721.1) | 29.6(13.9-53.6) | 5760.0(3672.9-8999.1) | 17.6(11.8-26.7) | -2.0(-1.9--2.1) |
| Zambia | 1800.3(1012.2-3106.5) | 19.9(11.9-33.6) | 1709.7(811.4-3272.4) | 11.7(5.2-22.8) | -2.5(-2.2--2.7) |
| Zimbabwe | 2000.0(1262.0-3596.5) | 21.1(13.4-38.6) | 4140.7(2566.0-7451.2) | 29.5(18.3-53.5) | 2.1(2.9-1.3) |
