# Supplementary figures and images for: Global, Regional, and National Burden of Myocarditis From 1990 to 2017: A Systematic Analysis Based on the Global Burden of Disease Study 2017
Source: Front Cardiovasc Med. 2021 Jul 2;8:692990. doi: 10.3389/fcvm.2021.692990 (PMC8284556; doi:10.3389/fcvm.2021.692990)

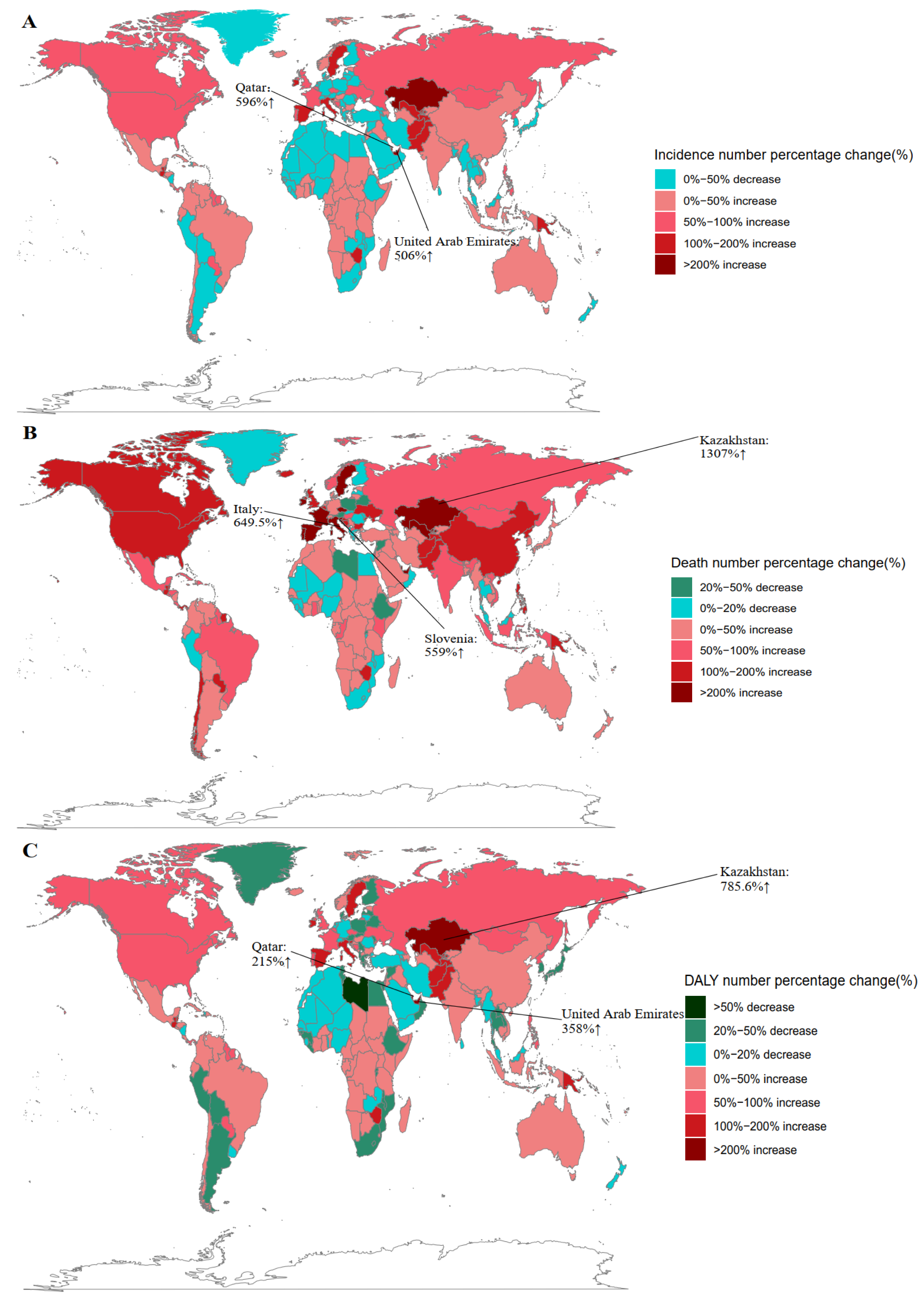

Supplement: Supplementary Figure 1 — The global disease burden of myocarditis for both genders in 195 countries and territories. (A) The relative change in incident cases of myocarditis between 1990 and 2017. (B) The relative change in myocarditis-related deaths between 1990 and 2017. (C) The relative change in DALY cases of myocarditis between 1990 and 2017. ASIR, age-standardized incidence rate; ASDR, age-standardized death rate; DALY, disability-adjusted life year; EAPC, estimated annual percentage change. [file Image_1.TIF]

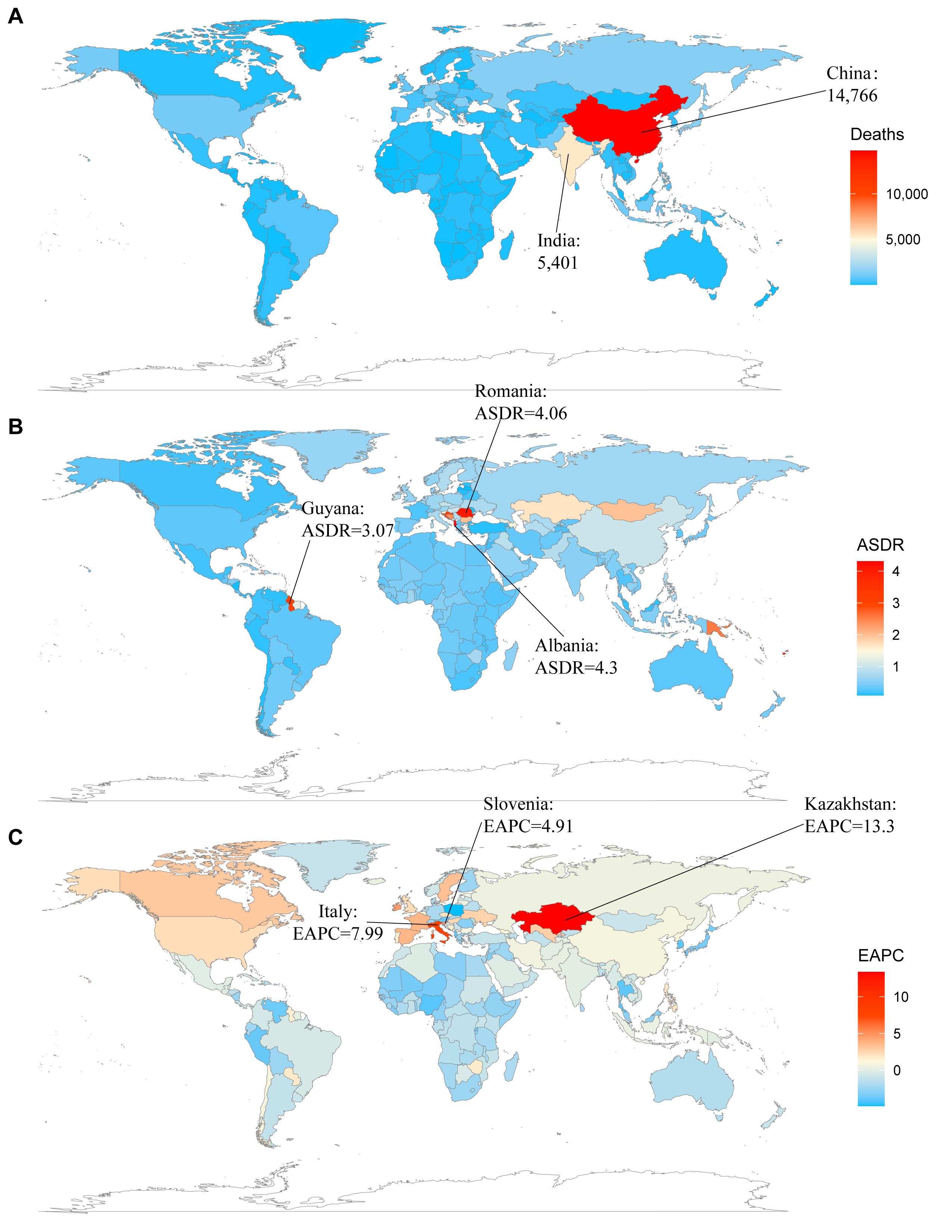

Supplement: Supplementary Figure 2 — The global death burden of myocarditis in 195 countries and territories. (A) The absolute number of myocarditis incidence cases in 2017. (B) The ASDR (per 100,000 persons) of myocarditis in 2017. (C) The EAPC of myocarditis ASDRs between 1990 and 2017. ASDR, age-standardized death rate; EAPC, estimated annual percentage change. [file Image_2.TIF]

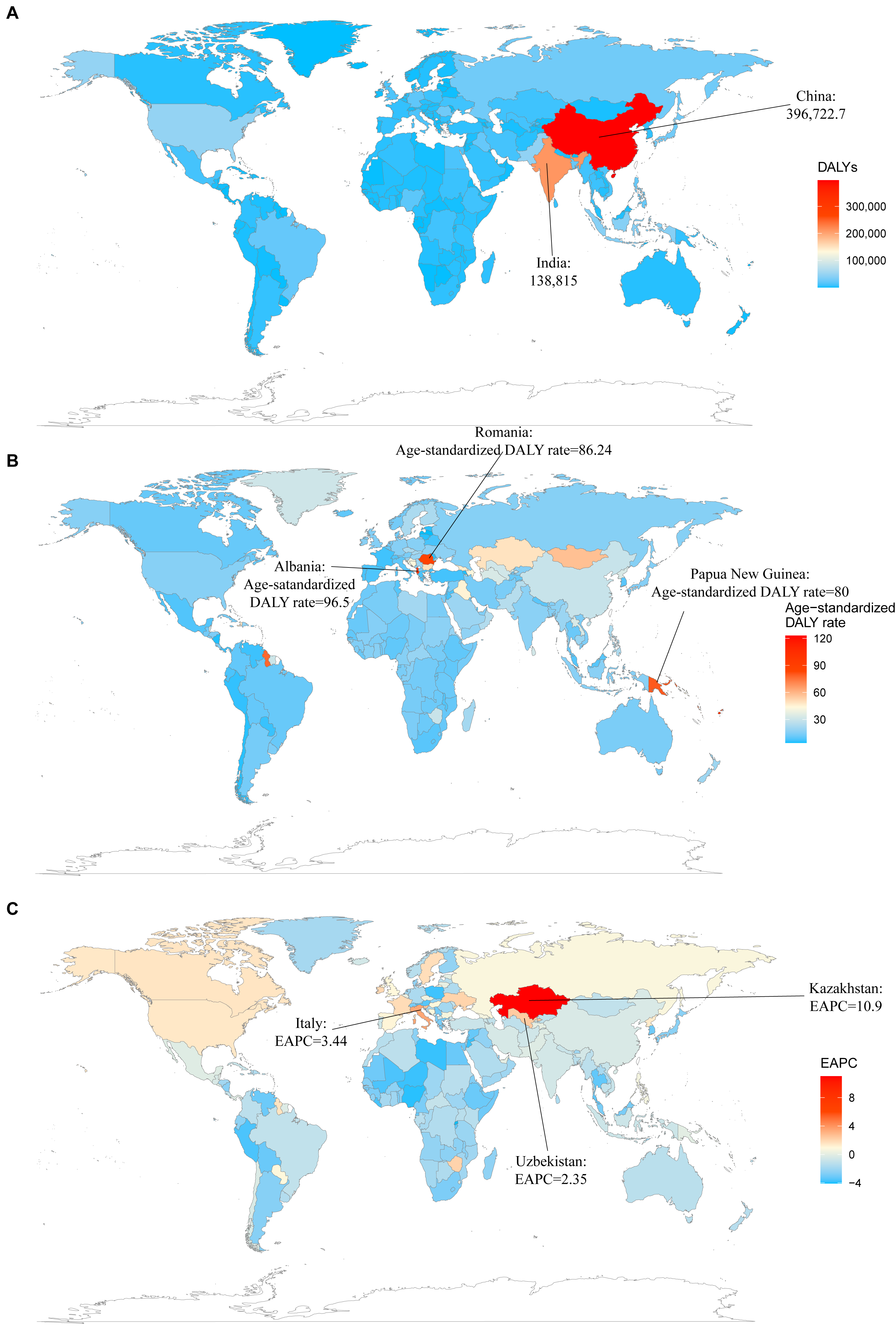

Supplement: Supplementary Figure 3 — The global DALY burden of myocarditis in 195 countries and territories. (A) The absolute number of myocarditis DALY cases in 2017. (B) The age-standard DALY rate (per 100,000 persons) of myocarditis in 2017. (C) The EAPC of myocarditis age-standardized DALY rate between 1990 and 2017. DALY, disability-adjusted life year; EAPC, estimated annual percentage change. [file Image_3.TIF]
